# Boosting understanding of Lassa Fever virus epidemiology: Field testing a novel assay to identify past Lassa Fever virus infection in blood and oral fluids of survivors and unexposed controls in Sierra Leone

**DOI:** 10.1371/journal.pntd.0009255

**Published:** 2021-03-31

**Authors:** Onome Akpogheneta, Steve Dicks, Donald Grant, Zainab Kanneh, Brima Jusu, Joseph Edem-Hotah, Lansana Kanneh, Foday Alhasan, Michael Gbakie, John Schieffelin, Samreen Ijaz, Richard Tedder, Hilary Bower

**Affiliations:** 1 Department of Infectious Disease Epidemiology, London School of Hygiene & Tropical Medicine, London, United Kingdom; 2 Blood Borne Virus Unit, Public Health England, Colindale, United Kingdom; 3 Microbiology Services, NHS Blood and Transplant, London, United Kingdom; 4 Kenema Government Hospital Lassa Fever Unit, Kenema, Sierra Leone; 5 Faculty of Nursing, University of Sierra Leone, Freetown, Sierra Leone; 6 Sections of Infectious Disease, Tulane University School of Medicine, New Orleans, Louisiana, United States of America; 7 Department of Infectious Disease, Imperial College, London, United Kingdom; 8 UK Public Health Rapid Support Team, London School of Hygiene & Tropical Medicine/Public Health England, London, United Kingdom; Center for Disease Control and Prevention, UNITED STATES

## Abstract

**Background:**

Despite identification 50 years ago, the true burden of Lassa Fever (LF) across Africa remains undefined for reasons including research focus on hospitalised patients, lack of validated field-feasible tools which reliably identify past infection, and the fact that all assays require blood samples making large-scale surveys difficult. Designated a priority pathogen of epidemic potential requiring urgent research by the World Health Organisation, a better understanding of LF sero-epidemiology is essential to developing and evaluating new interventions including vaccines. We describe the first field testing of a novel species-neutral Double Antigen Binding Assay (DABA) designed to detect antibodies to LF in plasma and oral fluid.

**Methodology/Principal findings:**

Paired plasma and oral fluid were collected in Sierra Leone from survivors discharged from Kenema Government Hospital Lassa Fever Unit between 1980 and 2018, and from controls recruited in Freetown in 2019. Epidemiological sensitivity and specificity of the DABA measured against historical diagnosis in survivors and self-declared non-exposed controls was 81.7% (95% CI 70.7%– 89.9%) and 83.3% (72.7%- 91.1%) respectively in plasma, and 71.8% (60.0%– 81.9%) and 83.3% (72.7%– 91.1%) respectively in oral fluid. Antibodies were identified in people infected up to 15 years and, in one case, 40 years previously. Participants found oral fluid collection easy and painless with 80% happy to give an oral fluid sample regularly.

**Conclusions/Significance:**

Given the difficulties of assay validation in a resource-limited setting, including unexpected exposures and diagnostics of varying accuracy, the new assay performed well in both plasma and oral fluid. Sensitivity and specificity are expected to be higher when case/control ascertainment is more definitive and further work is planned to investigate this. Even at the performance levels achieved, the species-neutral DABA has the potential to facilitate the large-scale seroprevalence surveys needed to underpin essential developments in LF control, as well as support zoonotic investigations.

## Author summary

Lassa Fever (LF) is a life-threatening rodent-borne viral haemorrhagic disease of epidemic potential particularly affecting some West African communities. Although it is estimated to cause 100,000–300,000 infections and 5,000 deaths annually, the true burden of the disease in the community is not well understood since research has largely focussed on hospitalised cases (thought to account for ~15% of infections) because of the difficulty of identifying mild or asymptomatic cases in the community. Gaining a better understanding of LF epidemiology, particularly the exposure and immunity status of populations in countries where the virus is endemic in the rodent hosts, is critical to developing control measures including vaccines which can protect against disease and/or reduce risk of epidemic transmission. A key step is to be able to identify who has been previously infected by virus, and therefore likely immune, and who remains susceptible by examining the prevalence of antibodies to the virus in the community. However, to date all tests require blood to be drawn which can be problematic for community participation as well as risky for collectors. This study reports on a project to develop and validate an antibody test that can be used with an easy-to-give oral fluid sample.

## Introduction

Lassa fever (LF) is an acute, potentially fatal, viral haemorrhagic zoonotic disease caused by Lassa virus (LASV) and one of eight diseases identified by the World Health Organisation (WHO) for urgent research and development as a priority pathogen of epidemic potential.[1] Although 80% of LASV infections are thought to be asymptomatic or cause mild disease and fatality overall is estimated around 1%, case fatality among the one in five who seek hospital care is 15% and rises to 80% without prompt treatment.[2,3]

Infection is acquired primarily via exposure to virus from rodent host excretions, fomites, or direct contact with infected hosts including humans.[3] Although the main rodent reservoir, the multimammate rat (*Mastomys natalensis)*, is found in many areas of Sub-Saharan Africa and LASV infection has been documented in large proportions of rodents,[4] infection in humans has been identified only in particular regions of West Africa, including Sierra Leone, Nigeria, Guinea, Liberia, Benin, Ghana and Mali.[3,5]

Although WHO estimates 100,000–300,000 LASV infections and 5,000 deaths annually, these figures are largely extrapolations from a single study carried out 35 years ago in Sierra Leone,[6] or from hospitalised cohorts unable to reflect the full burden of LASV in the community. Seroprevalence surveys that have been done suggest high numbers of undiagnosed infection in endemic and non-endemic areas.[7,8] A better understanding of LASV epidemiology, particularly the exposure and immunity status of populations in different parts of endemic countries, is critical to developing, targeting, and evaluating new interventions including vaccines.[9]

Two key obstacles can be identified: a dearth of validated field-feasible tools to identify past infection, and the fact that all assays require blood samples. Indeed, the drawing of blood has been found to be one of the most contentious issues in clinical trial processes, frequently arousing political, cultural, and social antipathy [10–13] as well as requiring considerable logistics to collect safely and store specimens.

A potential alternative is oral fluid. Found in the gingival crevice between teeth and gums, oral fluid contains traces of serum (usually 1–2μL/100 μL) which allows detection of antibodies usually difficult in saliva.[14] It is used routinely for HIV, and Hepatitis A, B and C diagnosis and to detect antibodies to viral infections such as mumps, measles, and rubella.[14–16] A highly sensitive and specific oral fluid Enzyme Linked Immunoassay (ELISA) developed to detect Ebola-specific antibodies has been used in community seroprevalence studies, evaluation of potential convalescent plasma donors, and to measure vaccine-induced antibodies.[17–20] Oral fluid assays are also being developed to identify people recovered from SARSCoV2.[21]

Oral-fluid sampling has major advantages over blood collection: it is minimally-invasive, more acceptable to subjects of all ages due to absence of pain and low or no perceived risk of contamination, does not need medically-trained personnel, and in non-COVID-19 pandemic times, safer for collectors, removing the risk of needle-stick injury and other collection and storage-related exposures.[22] The ability to carry out more comprehensive sampling due to higher acceptability would facilitate large-scale community-based research as well as offering a minimally-invasive option for tracking response to immunisation when trials are underway.

To investigate this alternative, we developed two novel assays: an IgG capture ELISA considered suitable for testing oral fluid and a double antigen binding assay (DABA) usually used with serum/plasma, but highly sensitive, adaptable for quantification and species neutral. Both assays were targeted to detect antibodies to Glycoprotein 2 (GP2). (S1 Text).

The assays were piloted in the UK using a panel of 20 stored plasma specimens from convalescent Sierra Leonean LF survivors, then taken to Sierra Leone to assess their performance characteristics in blood and oral fluid samples in an LASV-endemic country. This paper reports the results of a cohort study comparing the detection of antibodies in freshly-collected paired plasma and oral fluid samples from historically-confirmed LF survivors from the Kenema Government Hospital (KGH) Lassa Fever Unit (LFU), which receives and manages the majority of Sierra Leone’s LF cases,[23] and in individuals without known exposure.

## Methods

### Ethics statement

The study was approved by the Sierra Leone Ethics and Scientific Review Committee (SLESRC 28 Feb.2019) and the London School of Hygiene & Tropical Medicine Ethics Committee (Ref. No 16455). Written informed consent was obtained from all participants and from parents or guardians for children under 16 years. Written assent was obtained in addition from children aged 12–16 years.

### Participant recruitment

LF survivors were recruited prospectively as ‘cases’ if they were aged over 6 in June 2019, resident in Kenema District, and had been confirmed at the time of their illness to be LASV-positive according to the KGH LFU diagnostic algorithm which included positive results in one or more recombinant antigen, IgM or IgG ELISA, (ReLASV Pan-Lassa IGG/IGM & Antigen-capture ELISAs, Zalgen Labs, Germantown, MD), or clinically by a senior physician. Recruitment was limited to individuals who were confirmed LASV-positive between 2005–2018, i.e. recovered at least 1 year prior to this study, except for one insistent survivor with an admission date in 1980. Eligible individuals were identified by KGH LF outreach workers and invited to attend a group session in local language where they received oral and written information. Children were consented by a parent or guardian. A witness was present for those who were illiterate or requested support.

Selection of controls was challenging due to increasing uncertainty regarding the boundaries of Sierra Leone’s LASV endemic zone. We chose to recruit in Freetown, where LF is considered non-endemic and non-imported cases are reportedly rare, and from among students of the University of Sierra Leone Faculty of Nursing, partly because they were more likely to benefit from participation in a study which offered no direct health benefits through increased knowledge of LF and research methods, and partly because we speculated that they were more likely to understand the importance of declaring any potential LASV exposure. Control candidates were questioned about any known exposure to Lassa Fever and any presence in an “endemic” zone in their lifetime and those responding with a geographic or case-related risk of exposure were excluded. A LF and research seminar was offered regardless of recruitment or decision to participate. Study information was reviewed before obtaining written consent.

In both groups, anyone with a temperature above 37.9°C was excluded and advised to attend a clinic. All participants were paid the equivalent of US$10 to cover time and expenses.

### Data collection

Demographic data were collected electronically using LSHTM Open Data Kit software (S2 Text).[24] Survivors were asked to recall their LF experience, any exposure to LASV since their original infection, and recent experience of other illnesses; control candidates were excluded if they had exposure to LF as described above. After sampling, all participants were asked which form of sample collection they preferred.

Intended recruitment was 70 confirmed LF survivors and 70 unexposed controls to enable estimation of sensitivity and specificity with a precision of within +/- 10% based on the 95% confidence interval width for an observed estimate of 85%. Both study groups were a convenience sample of eligible individuals who were enrolled as they presented until the sample size was met.

### Sample collection

A 5ml venous sample was collected from each participant into labelled EDTA tubes (BD Vacutainer Hemogard) and kept in a cool box until the end of the day. On return, plasma was separated by centrifuge at 1,100g for 5 minutes and aliquots (200-500ul) prepared and stored in a –20°C freezer.

Use of the Oracol swab (Malvern Medical Developments, Worcester, UK) to collect oral fluid was demonstrated by the study team, then self-administered by participants, including by children, at times with adult support. Each swab was rubbed firmly along the gum line for 90 seconds, sealed, labelled, and kept in a cool box until transfer to a –20°C freezer.

Controls were recruited from 20 to 24 May, and survivors from 3 to 8 June and 27 to 28 September 2019. Sample analysis was performed in the London School of Hygiene & Tropical Medicine (LSHTM) Ebola Vaccine Trial Laboratory in Kambia, Sierra Leone from 1–3 October 2019.

### Laboratory methods

The DABA Total Antibody Assay uses microwell strips coated with LASV Glycoprotein 2 (GP2) antigen (Native Antigen Company, UK), and a horseradish peroxidase (HRP) GP2 antigen (GP2 Sheep Fc-Tag-HRP) (Native Antigen Company, UK) conjugate (MicroImmune, Clin Tech, UK) to detect all antibodies that specifically bind LASV GP2 antigen. For the DABA ELISA, plasma samples were diluted 1:5 in serum diluent (MicroImmune, Clin Tech, UK). Oral fluid samples were used without further dilution following initial preparation in TM. Diluted positive controls and study samples were transferred to the coated microwells (100 μL/well) and incubated for 60 minutes at 37°C. Microwells were washed five times with 300 μL/well of wash buffer (MicroImmune, Clin Tech, UK) using an automated ELISA plate washer. HRP conjugated LASV GP2 was added to the microwells (100 μL/well) and incubated at 37°C for 120 minutes. Microwells were again washed five times with 300 μL/well wash buffer. TMB substrate (MicroImmune, Clin Tech, UK) was added (100 μL/well) and incubated for 30 minutes followed by stop solution (MicroImmune, Clin Tech, UK) (50 μL/well). Microwells were read at 450 nm with an OD450.

To reduce the risk of aerosolised infectious particles in the lab, oral fluid sample preparation was done in a BSL2 cabinet. To generate the extract, 1ml transport medium (TM) (PBS, 10% FBS, 0.1% Tween-20, 0.5 μg/ml Amphotericin B, 0.25mg/ml Gentamicin) was added to the swab, agitated, and then squeezed out by rotating the swab against the wall of the tube, removing the need for a centrifuge. Aliquots of oral fluid in TM were prepared for direct use in assays without further dilution.

All samples were analysed in duplicate with the GP2 DABA and IgG Capture assays. Serial dilutions of positive control samples in serum diluent were used to derive a standard curve to determine arbitrary units (AU) to allow comparison of detected antibody levels in reactive samples. The laboratory technician was not blinded to cohort group. All laboratory processes are detailed in S1 Text: Laboratory Appendix.

Mixed method modelling, based on the distribution of the mean raw optical density (OD) of 69 negative sample pairs with results < 0.5 regardless of study group, was used to identify reactivity thresholds. One extreme outlier (borderline negative survivor) was dropped. Standard deviations were based on the sum of squares differences from the median, using negatives located above the median to counter the right skew in the data. Cut-offs were set at the median plus 4 standard deviations, giving a plasma cut-off of OD 0.12 and an oral fluid cut-off of OD 0.10.

### Analysis

Data were cleaned and categorical variables created where needed. Laboratory results were normalised using the ratio of the test OD to the cut-off so that normalised optical density (nOD) ≥1 is reactive. The G capture assay detected low levels of positive responses (0.07–0.54 in 1:50 dilution read at 450nm) in both samples types and exposure groups and is not discussed further here.

We assessed correlation between test runs and between plasma and oral fluid samples using Pearson’s Pairwise coefficients and evaluated sensitivity and specificity of the plasma and oral fluid assays using purported survivor and control status. Descriptive statistics are expressed as medians and Inter-Quartile Range (IQR) for continuous variables, and frequencies and percentages (n (%)) for categorical variables. Crude associations were tested with Fisher’s exact or Chi^2^ tests and adjusted using logistic regression. We performed sensitivity analyses to examine the effect on assay performance of using only laboratory-confirmed survivor cases, and of removing controls found to have presence in an endemic area. Confidence intervals were calculated with the Binomial exact method. Significance level was set at <0.05. STATA (StataCorp LLC, USA, V14.2) was used for all analyses. Overall results will be shared with authorities and participants either in-country or by video-conference. Individual results will not be shared to avoid any harm resulting from perceived immunity as it is not yet possible to interpret any protective effect of antibody presence.

## Results

### Study population characteristics

**Fig 1:**
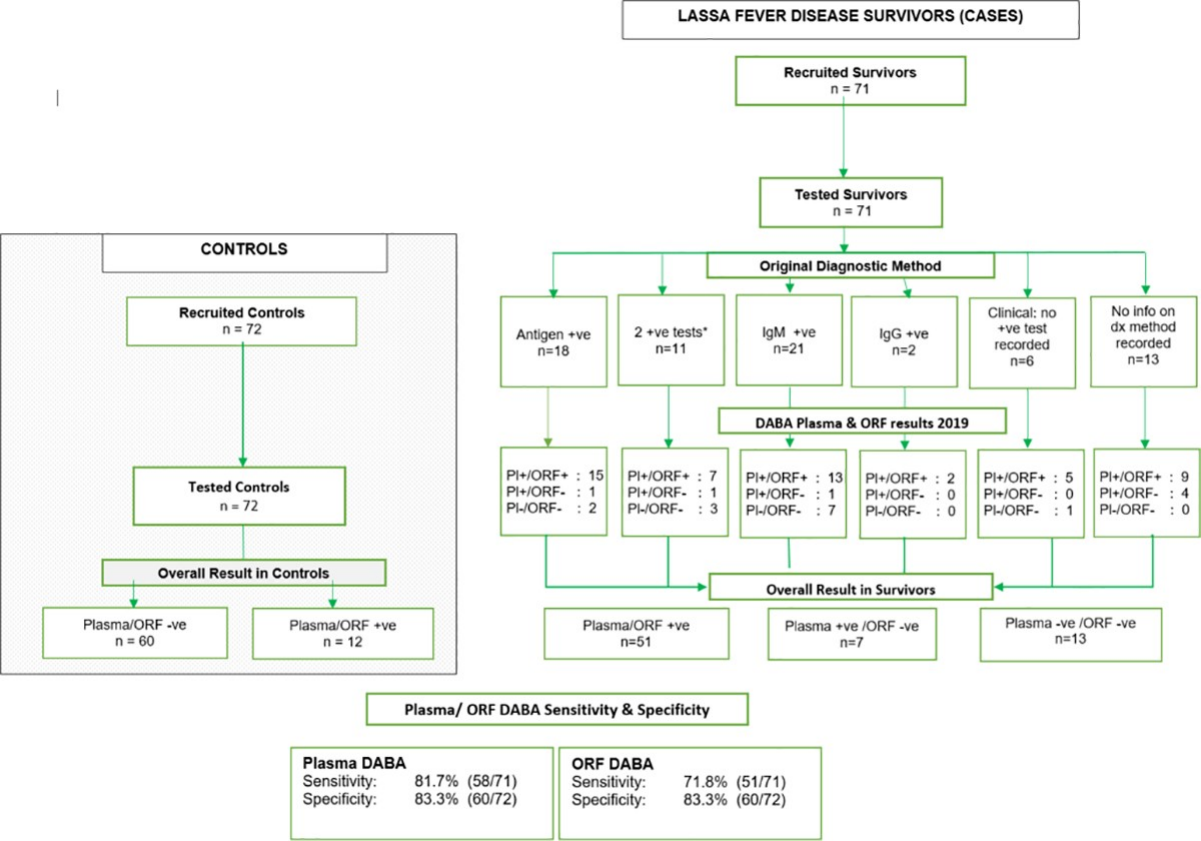
Flow diagram of study population and assay results.

The study population comprised 71 survivors from the KGH/LFU and 72 controls recruited as unexposed There were no exclusions at recruitment of controls as no candidate declared potential exposure. (Fig 1)

Controls had a narrower age range than survivors and were predominantly female and single due to recruitment at the nursing college. Eleven women (6 controls and 5 survivors) reported giving birth within the past 12 months but none were pregnant at the time of sampling. Twenty controls and 19 survivors reported having malaria in the past 12 months. No participant was a confirmed Ebola virus disease survivor. (Table 1)

**Table 1:** Characteristics of study population at the time of sampling.

	Survivors (n = 71)			Controls (n = 72)	
**Age in years** (median, IQR)	31.4	19.7–43.1	27.4		23.3–34.2
**Sex** (% female)	43	60.6%	65		90.3%
**Marital status**					
Single	28	39.4%	54		75.0%
**Occupation**					
HCW/Student nurse	1	1.4%	72		100%
Schoolchild/Student	25	35.7%	0		-
Farmer/Outdoor Worker	24	34.3%	0		-
Professional/Business	11	15.7%	0		-
Other	9	12.9%	0		-
**Current residence (district)**					
Freetown	0	-	67		93.1%
Kenema	71	100%	0		-
Bo	0	-	3		4.2%
Makeni	0	-	1		1.4%
Bonthe	0	-	1		1.4%
**Previous residence (district) if applicable**					
Freetown	0	-	63		92.7%
Kenema	38	90.5%	2		2.9%
Bo	2	4.8%	0		-
Kailahun	2	4.8%	0		-
Kono	0	-	1		1.5%
Moyamba	0	-	1		1.5%
Pujehun	0	-	1		1.5%
**Health (self-reported)**					
Pregnant at time of sampling	0	-	0		-
Delivered child <12months (female 16-49y)	5/32	15.6%	6/64		9.4%
Malaria illness < 12 months	19	27.1%	20		26.5%
Days since malaria illness (median, IQR)	403	341–449	70		8–95
# reporting a chronic illness	6	8.8%	1		1.4%
Ebola survivor (suspect)	1	1.5%	0		-

Survivors missing data: occupation, chronic disease, Ebola survivor, malaria illness, exposures—I participant; pregnancy/birth within 12m–1 participant; previous district—29 participants

Controls missing data: current residence 3 participants; previous residence—4 participants.

### Survivors

Survivors had experienced Lassa illness from 1 to 14 years ago, except for one who was ill almost 40 years ago. Sixty per cent had been admitted within 7 days of their symptom onset with the rest taking up to 31 days. Most survivors recalled mild or moderate illness (71.4%) at admission. (Tables 2 and S1)

Over the period, KGH employed different methods to diagnose LF. In our sample, 38.0% (27) were diagnosed using an IgM ELISA alone or in combination with an IgG ELISA (Zalgen, USA), 32.4% (23) were diagnosed using an antigen ELISA (Zalgen, USA) with or without an additional IgM ELISA and 13 (18.3%) had been admitted on clinical grounds. Of the remaining eight, two had only an IgG positive test, while for six only negative test results were recorded although LFU admission was confirmed in medical records and by outreach staff. (Table 2).

**Table 2:** Characteristics of the survivor cohort.

Age group (yrs)	Survivors (n = 71)	
<10	2	2.8%
11–15	9	12.7%
16–19	7	9.9%
20–29	16	22.5%
30–39	15	21.1%
40–49	11	15.5%
>50	11	15.5%
**Time since Lassa illness (median, IQR)**	5.5 years	4.4–6.3 years
**Time from onset to admission (median, IQR)**	7 days	5–9 days
**Method of diagnosis**		
Antigen(Ag) positive only	18	25.4%
Ag & IgM positive	5	7.0%
IgM & IgG positive	6	8.5%
IgM positive only	21	29.6%
IgG positive only	2	2.8%
All reported tests negative	6	8.5%
Clinical diagnosis only	13	18.3%
**Severity of symptoms at admission (recalled by participant at recruitment)**		
Mild	32	45.1%
Moderate	18	25.4%
Severe	20	28.2%
*Missing*	*1*	1.4%
**Type of exposure suspected (participant report)**		
Preparing rodents/small animals	3	4.3%
Caring for someone ill	3	4.3%
Contact with ill person not in your care	4	5.7%
Suspect case	10	14.3%
Don’t know	50	71.4%
**Lassa in household since discharge**	10	14.1%

### DABA performance

**Fig 2:**
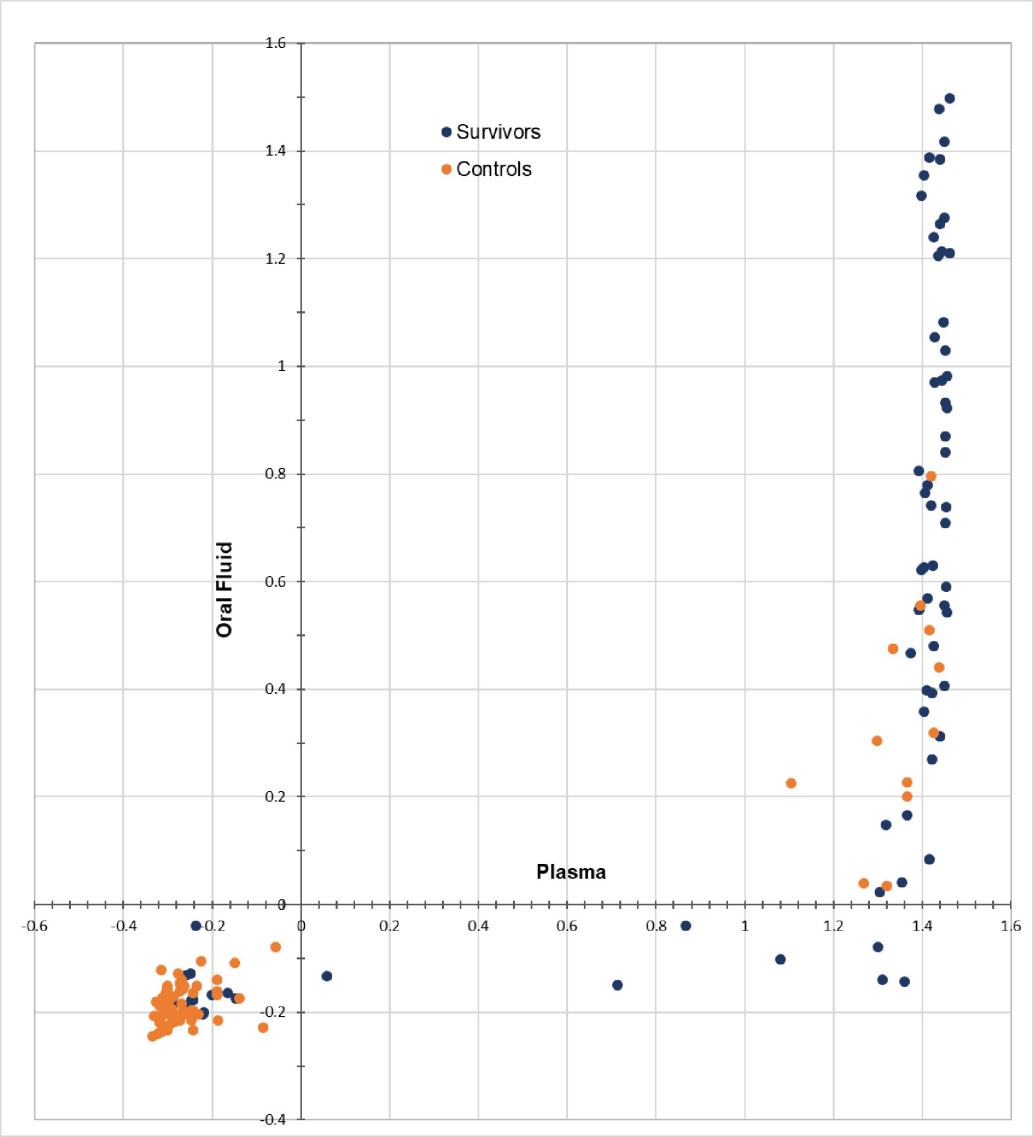
Distribution and performance characteristics of LASV DABA reactivity in plasma and oral fluid in survivor (n = 71) and control cohorts (n = 72) (nOD, log scale, reactive ≥ 0).

Duplicate runs of the DABA were tightly correlated in both plasma and oral fluid (R^2^ 0.98, & 0.87 respectively, p <0.001), showing excellent reproducibility. Of the 71 survivors, 58 (81.7%) were sero-positive for Lassa antibodies on plasma DABA, and 71.8% (51/71) on oral fluid DABA (Fig 2 and Table 3). This included five of the six samples from individuals recorded as being negative on all diagnostic tests whose study samples were strongly reactive on DABA in both plasma and oral fluid. Thirteen survivors were unreactive in both plasma and oral fluid. Of the 72 controls, 60 (83.3%) were Lassa antibody sero-negative in plasma and oral fluid; the remaining 12 individuals were sero-positive.

**Table 3:** Sensitivity and Specificity of the LASV DABA test in plasma and oral fluid.

	Reference group	
**Outcome DABA PLASMA**	**Survivor**	**Control**
Plasma +	58	12
Plasma -	13	60
Total	71	72
**Sensitivity (95% CI)**	**58/71: 81.7% (70.7–89.9%)**	
**Specificity (95% CI)**		**60/72: 83.3% (72.7–91.1%)**
**Outcome DABA ORAL FLUID**	**Survivor**	**Control**
ORF +	51	12
ORF -	20	60
Total	71	72
**Sensitivity (95% CI)**	**51/71: 71.8% (60.0–81.9%)**	
**Specificity (95% CI)**		**60/72: 83.3% (72.7–91.1%)**

Raw OD values read at OD 450nm ranged from 0.062 to 3.49 in plasma, and from 0.063 to 3.14 in oral fluid. Normalised values (nOD) ranged from 0.517 to 29.98 in plasma and from 0.625 to 31.44 in oral fluid (reactivity ≥1).

**Fig 3:**
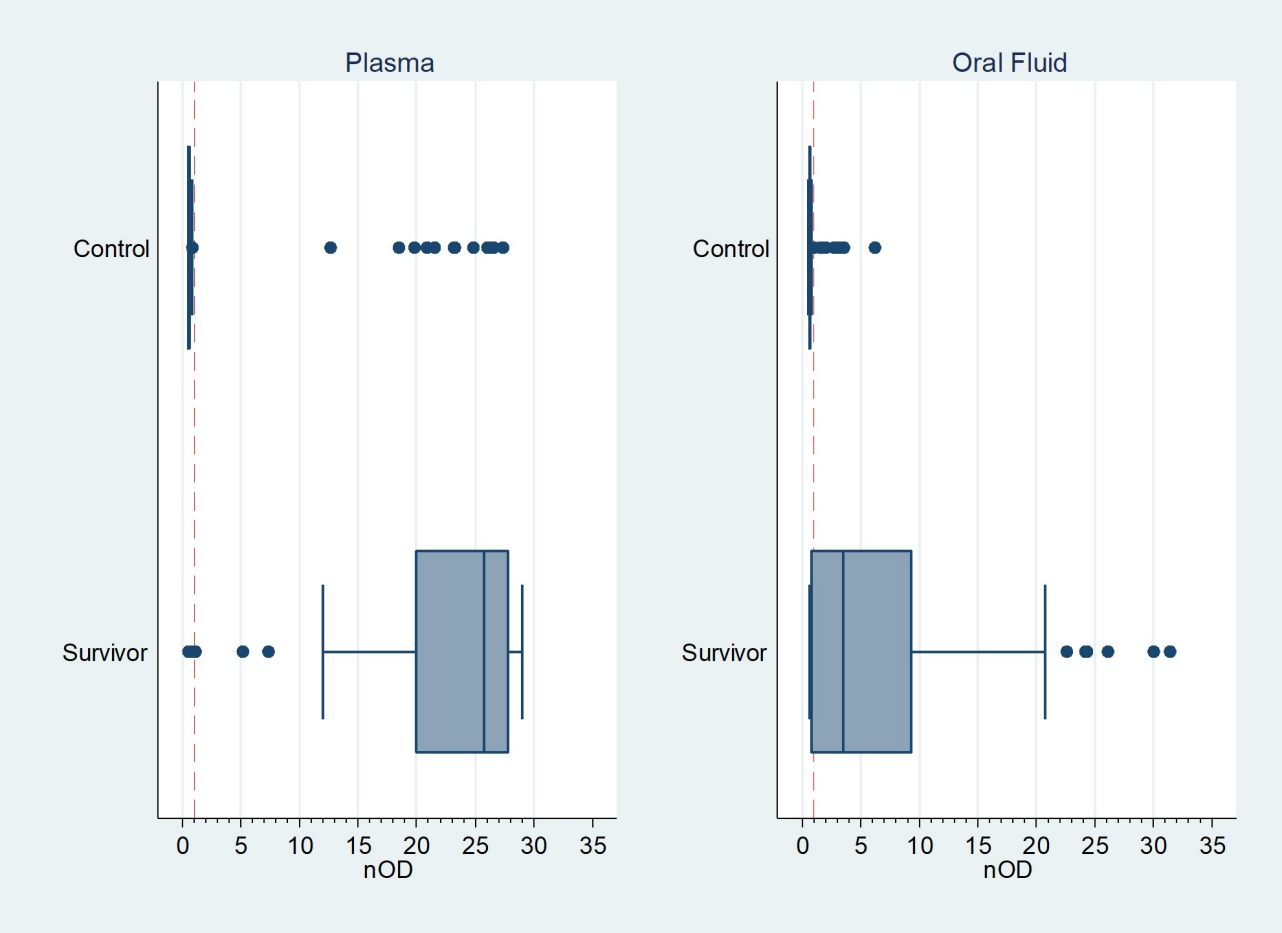
Distribution of LASV DABA assay reactivity in plasma and oral fluid samples in survivors (n = 71) and control (n = 72) cohorts. Normalised cut-off of 1: results above the red dotted line are positive on DABA. Vertical lines show median and inter-quartile range.

**Fig 4:**
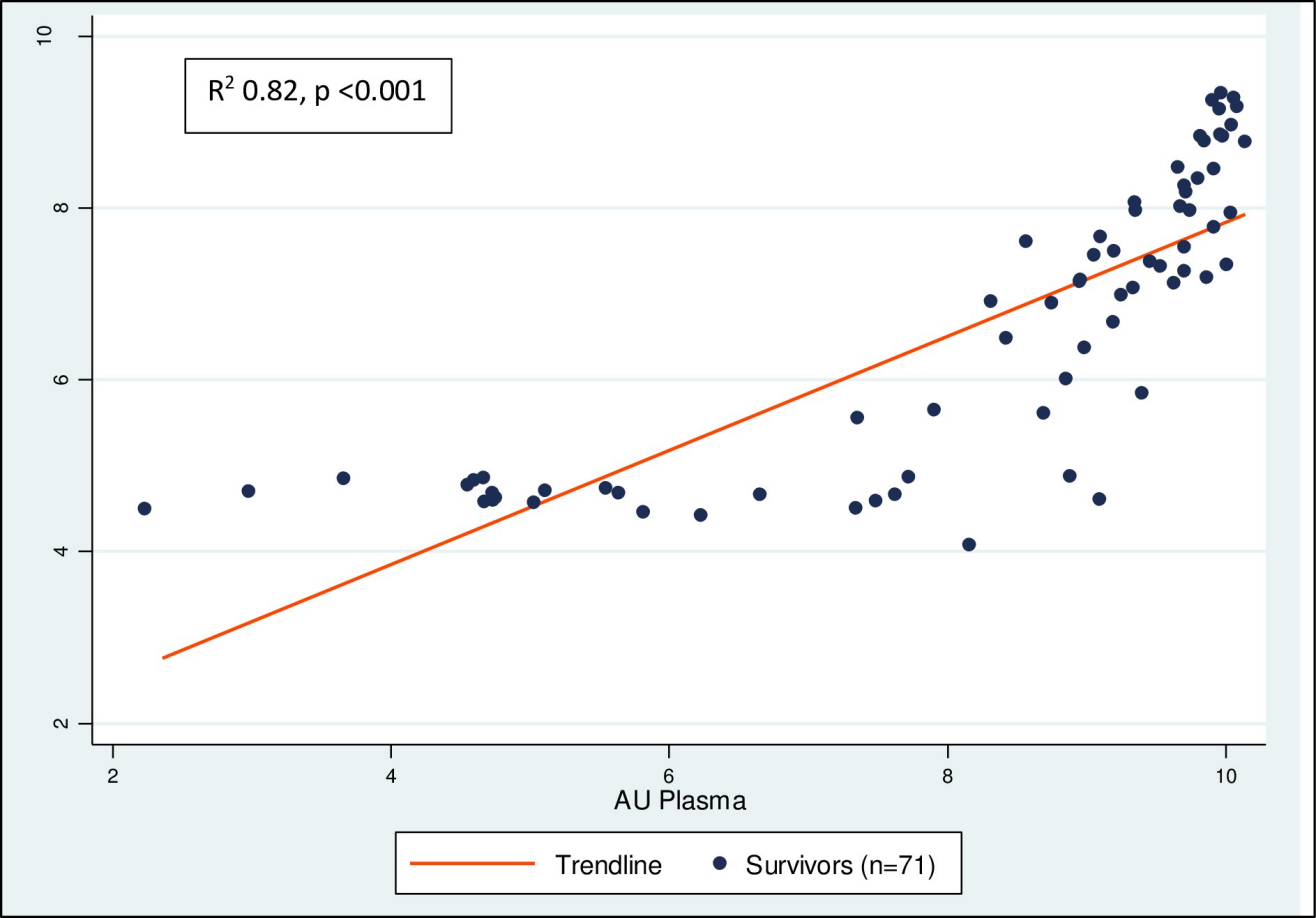
Concordance of antibody levels in plasma and oral fluid samples measured by arbitrary unit (AU) (survivors only, log scale).

Reactive and non-reactive samples were clearly separated in both plasma (median nOD: reactive 26.2, IQR 23.3–27.8; unreactive 0.53, IQR 0.50–0.57) and oral fluid (median nOD: reactive 4.23, IQR 2.47–10.68; unreactive 0.66, IQR 0.62–0.70) (Figs 2 and 3). Arbitrary units were quantified to estimate antibody level detected in samples and showed high concordance between the paired plasma and oral samples of survivors. (R2 0.82, p <0.001, Fig 4).

Under the study conditions, the sensitivity and specificity of the LASV DABA in plasma was 81.7% (95% CI 70.7%–89.9%) and 83.3% (72.7%- 91.1%) respectively and sensitivity and specificity of the LASV DABA in oral fluid was 71.8% (60.0%– 81.9%) and 83.3% (72.7%–91.1%) respectively. (Fig 2 and Table 3) Interpretation of these results, given the later discovery of undeclared exposure among controls and evolving understanding of the accuracy of original diagnostic methods over time, is explored in the discussion.

**Fig 5:**
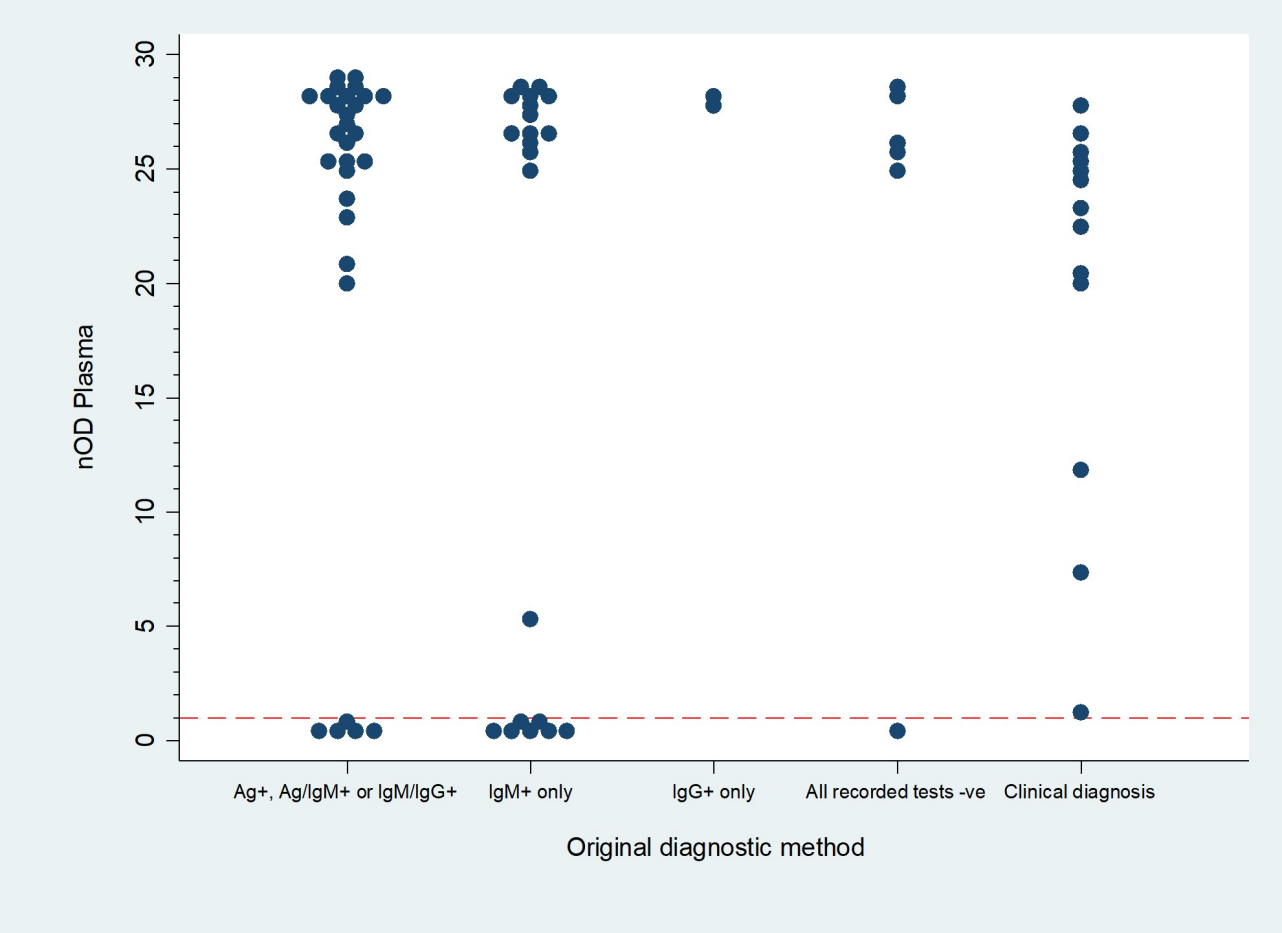
Distribution of Plasma DABA results by original diagnostic method of survivors (n = 71). Normalised cut-off of 1: results above the line are positive on DABA. Ag+: ReLASV Antigen ELISA positive. IgM+: ReLASV IgM ELISA positive. IgG+: ReLASV IgG ELISA positive. Ag+, Ag/IgM+, IgM/IgG+: combinations of positive test.

DABA plasma and oral fluid findings were more likely to concur with the results of original testing where diagnosis was based on the combination of a positive antigen and a positive IgM test (OR 20, p = 0.02) (Fig 5 and Table 4), though the relationship was lost when adjusted for year of testing (aOR 8.4, p = 0.08) due to the variability of diagnostic tests used over time.

**Table 4:** Distribution of DABA results in plasma and oral fluid by method used to confirm diagnosis at onset (see also Fig 5).

Original diagnostic method		DABA plasma +		DABA Oral Fluid +	
**Ag+, Ag/IgM+ or IgM/IgM+**	29 (40.8%)	24	*82*.*8%*	22	*75*.*9%*
**IgM+ only**	21 (29.6%)	14	*66*.*7%*	13	*61*.*9%*
**IgG+ only**	2 (2.8%)	2	*100*.*0%*	2	*100*.*0%*
**All recorded tests neg**	6 (8.5%)	5	*83*.*3%*	5	*83*.*3%*
**Clinical diagnosis**	13 (18.3%)	13	*100*.*0%*	9	*69*.*2%*
**Total**	71 (100.0%)	58	*81*.*7%*	51	*71*.*8%*

### Effect of time since illness and subsequent exposure on reactivity

**Fig 6:**
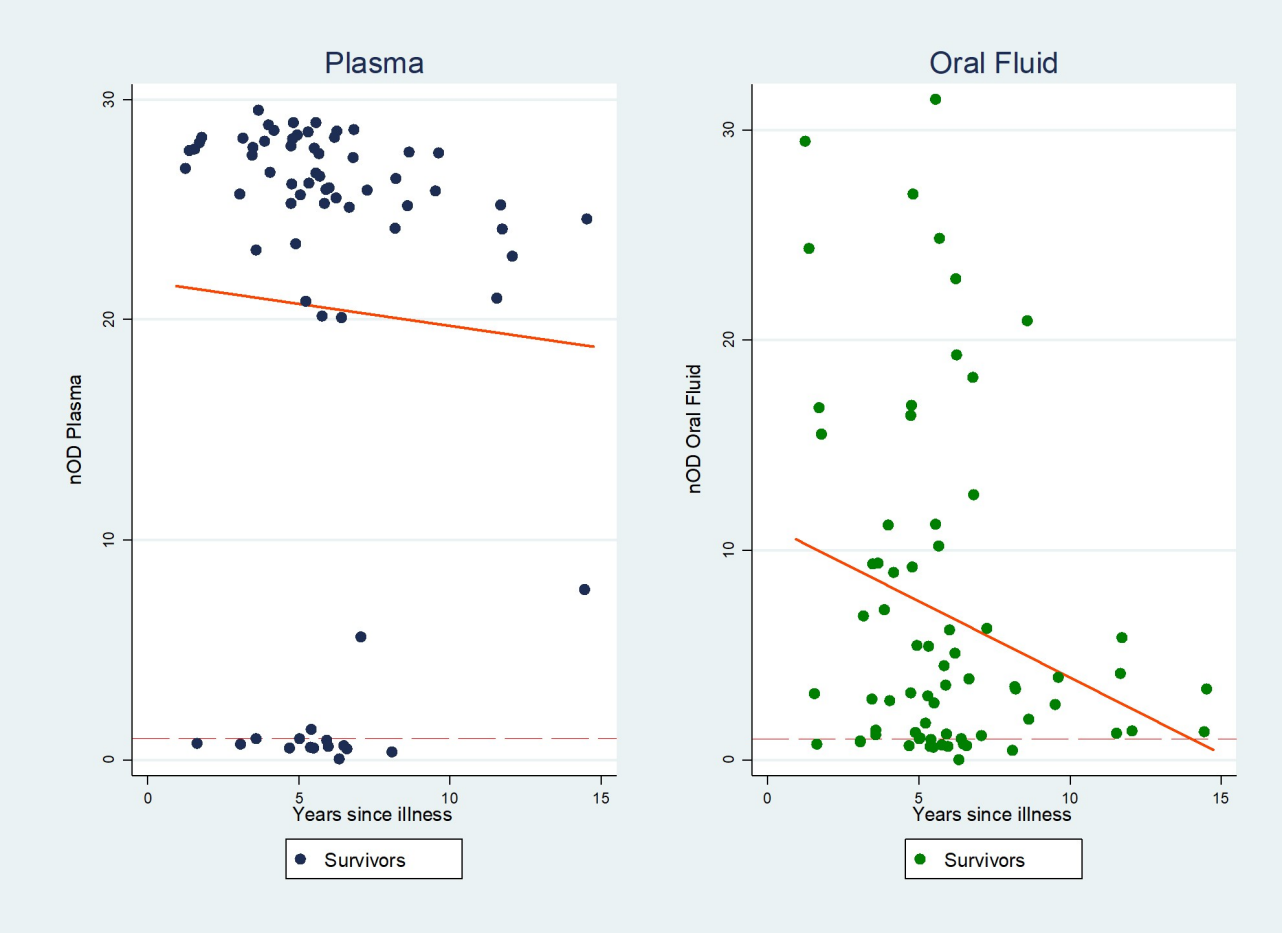
Association of level of antibody reactivity with time (n = 70). Trendline Normalised cut-off of 1: results above the red dotted line are positive on DABA. Outlier at 40 years excluded.

Antibody presence showed only a small drop off with time since illness (Fig 6). Reactivity in both plasma and oral fluid was slightly negatively correlated (Coef. -0.23 p 0.38, -0.35, p 0.07 respectively) with time elapsed since participants were ill, becoming significant when unreactive samples were excluded (Coef. -0.41 plasma p 0.001; -0.18 oral fluid p 0.03). Five of 10 survivors who reported another case of Lassa in their household since their discharge had higher than mean results but not significantly different (p 0.4) to those without recalled household contact. The participant with onset in 1980 showed definitive sero-positivity on plasma (nOD 12.03) though substantially lower than the mean of nOD 25.77 and was not reactive on oral fluid (nOD 0.79). Survivor participants under 20 years old were more likely to be reactive on both analytes (OR 12.2, p <0.001) but no other demographic, exposure, or health factor, including malaria within the past 12 months, was associated with sero-positivity.

### Discordant results

Thirteen survivor and 12 ‘unexposed’ control participants had DABA results discordant with their recruitment group.

Among the 13 survivors, those who were unexpectedly DABA sero-negative were more likely to be under 20 years old (adjusted Odds Ratio (aOR) 18.4, p 0.001), and to have been originally diagnosed by an IgM test only (aOR 6.45, p 0.059) between 2013 and 2015, (aOR 1.8, p 0.713). Seven of the 13 had only an IgM positive test recorded, five had one or two positive Ag/IG tests, and one had no recorded positive test (Fig 5). Of all other variables, including symptom severity, only abdominal pain was associated (p 0.02) with a negative DABA result in survivors. All 13 had antibody reactivity close to the mean for negative samples in both their plasma and oral fluids.

Among the 12 controls who were unexpectedly DABA sero-positive, significantly more were male (aOR 11.1, p 0.015) and over 40 (aOR 9.9, p 0.039). They were also slightly though non-significantly more likely to have spent time in a Lassa-endemic area (aOR 1.4, p 0.746): 2 of the 12 were found to have previously spent time in the Lassa endemic zones of Kenema and Kono. All 12 were strongly sero-positive in plasma including five with ODs over the mean.

## Discussion

We have developed and validated a new method of identifying past infection with Lassa Fever virus using blood and oral fluid specimens in Sierra Leone where the disease has a substantial impact on population health. Despite challenges in confirming survivor and exposure status, our findings have shown the novel Lassa Double Antigen Binding Assay to have good diagnostic performance in both specimen types in people infected up to 15 years ago and, in one case, 40 years previously.

Under the study conditions, the sensitivity and specificity of the LASV DABA in plasma was 81.7% and 83.3%respectively, and the sensitivity and specificity of the LASV DABA in oral fluid was 71.8% and 83.3% respectively. We used a mixed methods modelling approach to set the positivity cut-off to hone performance, but it is worth noting that performance was not significantly different when a standard field kit cut-off (mean of the plate negative controls plus 0.1) was used (plasma sensitivity 80.3%, specificity 83.3%; oral fluid sensitivity 64.8%, specificity 87.5%).

Our findings, particularly those using oral fluid sampling, have important implications for the epidemiological research needed to clarify the burden of Lassa Fever infection and facilitate the development of candidate vaccines where understanding population serostatus is a critical issue.[25,26] The ability to sample for a viral haemorrhagic fever without invasive blood-draw, thereby reducing risk of community reluctance and biosecurity collection hazards, opens the possibility of performing the largescale seroprevalence surveys needed to clarify the geographical reach, range of severity, and transmission dynamics of LASV, not only in countries where there is known human incidence of disease, but also in countries where the virus is endemic in rodent hosts.[27]

Although additional precautions would be needed during the COVID-19 era, the use of oral fluid testing would support cost-effective investigations, alone or as a screen for a confirmatory plasma assay, to reduce costs and participant burden for assessing prevalence prior to vaccine trials. Use of oral fluid testing will also facilitate the longitudinal research needed to establish the level and duration of protective immunity, since the majority of study participants said they preferred to give an oral fluid sample than a blood sample, most commenting that it was easier and painless, and 80% said they would be happy to give an oral fluid sample regularly for research. Of those that preferred to give blood, several felt that blood tests would give more thorough information–a point to keep in mind when creating public information around oral fluid sampling.

It must also be highlighted that the DABA assay, uniquely, is species-neutral, offering a powerful tool for investigation of the rodent reservoir and the zoonotic distribution of LASV along-side human surveys.

Measuring the performance of the DABA assay was affected by some important limitations in the study environment. These include the need to rely on original diagnostic tests and medical records to identify LF survivors, the lack of a feasible benchmark test for past infection, and the difficulty of obtaining truly unexposed controls in Sierra Leone. There may also have been recall bias regarding possible re-exposure, and potential for cross-reactivity with other arenaviruses, although this was substantially reduced due to the double-binding of the assay.

While the KGH diagnostic protocol for LF has evolved through the institution’s substantial work on LF laboratory science and now involves a more reliable combination of antigen and antibody tests (the test combination that correlated more closely with DABA results),[28] only 23 of the 71 survivors recruited for our study were originally diagnosed with an assay or assay combination with sensitivity greater than 88%.[29,30] The remainder were diagnosed with assays (IgM, IgG, or IgM/IgG) with reported sensitivity of 26–57%,[28] or admitted on clinical grounds. As seven of the 13 discordant survivors were initially diagnosed with an assay with high risk of false positivity, it is possible that DABA sensitivity is underestimated.

Alternate analysis using only survivors confirmed with an antigen test or 2 different assays (24/29), showed a non-significant increase in sensitivity of the plasma DABA from 81.7% (CI 70.7%–89.9%) to 82.8% (CI 64.2%- 94.1%), and of oral fluid from 71.8% (CI 60.0%– 81.9%) to 75.9% (CI 56.5%– 89.7%).

Testing assays in the population of the country is good practice. But despite efforts to exclude people with Sierra Leone endemic zone contact from the control group, unknown or undeclared exposure could not be ruled out, not least because the endemic zone borders in Sierra Leone have become less distinct. In 2014, one in seven Ebola-negative patients presenting to the Western Area Rural Hospital of Jui (20 km from Freetown) were Lassa positive.[31] Some non-endemic rural areas have been reported to have seroprevalence as high as 88%[32] while small outbreaks in the Northern Region have been sparked by in-country travellers from endemic districts.[33] All raise the possibility of unwitting exposure and un-noticed infection among our controls which is likely to have led to underestimation of DABA specificity. For example, alternate analysis in which we excluded the eight participants later discovered to have had previous presence in endemic areas increased specificity from 83.3% to 84.4% in both specimen types.

Finally, we were not able to measure the performance of the new assay against an reliable benchmark due to the difficulty of effectively running the only existing validated IgG serological test (US CDC IgG ELISA[30]) in a resource-limited setting.

All these constraints will, however, be addressed shortly when the plasma DABA is assessed in the Foundation for Innovative New Diagnostics (FIND) Independent Evaluation of Lassa Fever Virus Serology. Using the US CDC IgG ELISA as the benchmark and with blinded laboratory staff, the evaluation will measure candidate assays against case samples from Sierra Leone and Nigeria, thereby covering all 4 LASV lineages, and against control samples from an African country where there has, to date, been no evidence of LASV in humans (Gabon). As only stored blood samples are available, the evaluation will not assess the oral fluid DABA, but given the tight concordance of the two specimen types, we hope the findings will reinforce the benefits of using this novel approach to improve knowledge and assist the development of Lassa Fever prevention and control measures.

## Supporting information

S1 STARD Checklist (DOCX)

S1 Text Laboratory protocols. A. In-country laboratory validation processes. B. DABA Total Antibody Assay Information for Users.
(DOCX)

S2 Text Study tools. A. Protocol. B. Consent/Assent form. C. Questionnaires and control eligibility check list. D. Patient information leaflet.
(DOCX)

S1 Table Symptoms at admission recalled by survivors at time of recruitment. (DOCX)
