# Supplementary material for: Boosting understanding of Lassa Fever virus epidemiology: Field testing a novel assay to identify past Lassa Fever virus infection in blood and oral fluids of survivors and unexposed controls in Sierra Leone
Source: PLoS Negl Trop Dis. 2021 Mar 31;15(3):e0009255. doi: 10.1371/journal.pntd.0009255 (PMC8041174; doi:10.1371/journal.pntd.0009255)
Supplement: S1 STARD Checklist — (DOCX) [file pntd.0009255.s001.docx]

|  | **Section & Topic** | **No** | **Item** | **Reported on page #** |
| --- | --- | --- | --- | --- |
|  |  |  |  |  |
|  | **TITLE OR ABSTRACT** |  | **A novel approach to boost understanding of Lassa Fever virus epidemiology: field validation of a new assay to identify past Lassa Fever virus infection in blood and oral fluids of survivors and unexposed controls in Sierra Leone.**  **Corresponding author: Hilary Bower, hilary.bower@lshtm.ac.uk** |  |
|  |  | **1** | Identification as a study of diagnostic accuracy using at least one measure of accuracy(such as sensitivity, specificity, predictive values, or AUC)  L2: Field validation of a new assay to identify past Lassa Fever virus infection in blood and oral fluids  L68: (Abstract): Epidemiological sensitivity and specificity of the DABA measured against historical diagnosis in survivors and self-declared non-exposed controls was…. | Please see line numbers in left hand column |
|  | **ABSTRACT** |  |  |  |
|  |  | **2** | Structured summary of study design, methods, results, and conclusions  (for specific guidance, see STARD for Abstracts)  L55-79: formatted according to PLoS NTD abstract style : Background, Methodology/Principal findings. Conclusions/Significance |  |
|  | **INTRODUCTION** |  |  |  |
|  |  | **3** | Scientific and clinical background, including the intended use and clinical role of the index test  L104: Although WHO estimates 100,000-300,000 LASV infections and 5,000 deaths annually, these figures are largely extrapolations from a single study carried out 35 years ago in Sierra Leone^6^, or from hospitalised cohorts unable to reflect the full burden of LASV in the community. Seroprevalence surveys that have been done suggest high numbers of undiagnosed infection in endemic and non-endemic areas^7,8^ A better understanding of LASV epidemiology, particularly the exposure and immunity status of populations in different parts of endemic countries, is critical to developing, targeting, and evaluating new interventions including vaccines.^9^  Two key obstacles can be identified: a dearth of validated field-feasible tools to identify past infection, and the fact that all assays require blood samples. Indeed, the drawing of blood has been found to be one of the most contentious issues in clinical trial processes, frequently arousing political, cultural, and social antipathy^10-13^ as well as requiring considerable logistics to collect safely and store specimens.  A potential alternative is oral fluid. Found in the gingival crevice between teeth and gums, oral fluid contains traces of serum (usually 1-2µL/100 µL) which allows detection of antibodies usually difficult in saliva.^14^ It is used routinely for HIV, and Hepatitis A, B and C diagnosis and to detect antibodies to viral infections such as mumps, measles, and rubella.^14-16^ A highly sensitive and specific oral fluid Enzyme Linked Immunoassay (ELISA) developed to detect Ebola-specific antibodies has been used in community seroprevalence studies, evaluation of potential convalescent plasma donors, and to measure vaccine-induced antibodies.^17-20^ Oral fluid assays are also being developed to identify people recovered from SARSCoV2.^21^  Oral-fluid sampling has major advantages over blood collection: it is minimally-invasive, more acceptable to subjects of all ages due to absence of pain and low or no perceived risk of contamination, does not need medically-trained personnel, and in non-COVID-19 pandemic times, safer for collectors, removing the risk of needle-stick injury and other collection and storage-related exposures.^22^ The ability to carry out more comprehensive sampling due to higher acceptability would facilitate large-scale community-based research as well as offering a minimally-invasive option for tracking response to immunisation when trials are underway.  L129: To investigate this alternative, we developed two novel assays: an IgG capture ELISA considered suitable for testing oral fluid and a double antigen binding assay (DABA) usually used with serum/plasma, but highly sensitive, adaptable for quantification and species neutral. Both assays were targeted to detect antibodies to Glycoprotein 2 (GP2). |  |
|  |  | **4** | Study objectives and hypotheses  L129: ….developed two novel assays: an IgG capture ELISA considered suitable for testing oral fluid and a double antigen binding assay (DABA) usually used with serum/plasma, but highly sensitive, adaptable for quantification and species neutral.  L134: ….field-validation in Sierra Leone with a cohort study using freshly-collected paired plasma and oral fluid samples from historically-confirmed LF survivors from the Kenema Government Hospital (KGH) Lassa Fever Unit (LFU), which receives and manages the majority of Sierra Leone’s LF cases,23 and individuals without known exposure. This paper reports the results of this field validation study. |  |
|  | **METHODS** |  |  |  |
|  | *Study design* | **5** | Whether data collection was planned before the index test and reference standard  were performed (prospective study) or after (retrospective study)  L141: Participants were recruited prospectively. Cases were LF survivors aged over 6 in June 2019, resident in Kenema District, who had a confirmed diagnosis from KGH LFU based on positive results in one or more recombinant antigen, IgM or IgG ELISA, (ReLASV® Pan-Lassa IGG/IGM & Antigen-capture ELISAs, Zalgen Labs, Germantown, MD), or clinically by a senior physician. Recruitment was limited to cases confirmed between 2005-2018,  L149 Selection of controls was challenging due to increasing uncertainty regarding the boundaries of Sierra Leone’s LASV endemic zone. We chose to recruit in Freetown, where LF is considered non-endemic and non-imported cases are reportedly rare, and from among students of the University of Sierra Leone Faculty of Nursing…… |  |
|  | *Participants* | **6** | Eligibility criteria  L141 : LF survivors aged over 6 in June 2019, resident in Kenema District, who had a confirmed diagnosis from KGH LFU based on positive results in one or more recombinant antigen, IgM or IgG ELISA, (ReLASV® Pan-Lassa IGG/IGM & Antigen-capture ELISAs, Zalgen Labs, Germantown, MD), or clinically by a senior physician. Recruitment was limited to cases confirmed between 2005-2018,  L150: We chose to recruit in Freetown, where LF is considered non-endemic and non-imported cases are reportedly rare, and from among students of the University of Sierra Leone Faculty of Nursing, partly because they were more likely to benefit from participation in a study which offered no direct health benefits through increased knowledge of LF and research methods, and partly because we speculated that they were more likely to understand the importance of declaring any potential LASV exposure. Control candidates were questioned about any known exposure to Lassa Fever and any presence in an “endemic” zone in their lifetime and those responding with a geographic or case-related risk of exposure were excluded. |  |
|  |  | **7** | On what basis potentially eligible participants were identified  (such as symptoms, results from previous tests, inclusion in registry)  See section 6 |  |
|  |  | **8** | Where and when potentially eligible participants were identified (setting, location and dates)  See section 6 |  |
|  |  | **9** | Whether participants formed a consecutive, random or convenience series  L171: Both study groups were a convenience sample of eligible individuals who were enrolled as they presented until the sample size was met. |  |
|  | *Test methods* | **10a** | Index test, in sufficient detail to allow replication  Supporting Information 1 : complete development process and laboratory methods are detailed |  |
|  |  | **10b** | Reference standard, in sufficient detail to allow replication  L111: Two key obstacles can be identified: a dearth of validated field-feasible tools to identify past infection, and the fact that all assays require blood samples.  L134: field-validated in Sierra Leone with a cohort study using freshly-collected paired plasma and oral fluid samples from historically-confirmed LF survivors from the Kenema Government Hospital (KGH) Lassa Fever Unit (LFU), which receives and manages the majority of Sierra Leone’s LF cases,^23^ and individuals without known exposure.  L 381: Finally, we were not able to measure the performance of the new assay against an reliable benchmark due to the difficulty of effectively running the only existing validated IgG serological test ( US CDC IgG ELISA30) in a field study setting. |  |
|  |  | **11** | Rationale for choosing the reference standard (if alternatives exist)  See section 10B |  |
|  |  | **12a** | Definition of and rationale for test positivity cut-offs or result categories  of the index test, distinguishing pre-specified from exploratory  L194: Mixed method modelling, based on the distribution of the mean raw optical density (OD) of 69 negative sample pairs with results < 0.5 regardless of study group, was used to identify reactivity thresholds. One extreme outlier (borderline negative survivor) was dropped. Standard deviations were based on the sum of squares differences from the median, using negatives located above the median to counter the right skew in the data. Cut-offs were set at the median plus 4 standard deviations, giving a plasma cut-off of OD 0.12 and an oral fluid cut-off of OD 0.10. |  |
|  |  | **12b** | Definition of and rationale for test positivity cut-offs or result categories  of the reference standard, distinguishing pre-specified from exploratory  See above |  |
|  |  | **13a** | Whether clinical information and reference standard results were available  to the performers/readers of the index test  L193: The laboratory technician was not blinded to cohort status, but clinical and epidemiological information were not available. |  |
|  |  | **13b** | Whether clinical information and index test results were available  to the assessors of the reference standard  Not applicable – see section 10b |  |
|  | *Analysis* | **14** | Methods for estimating or comparing measures of diagnostic accuracy  L206: correlation between test runs and between plasma and oral fluid samples using Pearson’s Pairwise coefficients and evaluated sensitivity and specificity of the plasma and oral fluid assays in a field setting using purported survivor and control status. |  |
|  |  | **15** | How indeterminate index test or reference standard results were handled  There were no indeterminate results: one cell contamination was mitigated by the use of the first uncontaminated test result as the ‘mean’ instead of creating a mean from the two duplicated tests as was done with all other tests |  |
|  |  | **16** | How missing data on the index test and reference standard were handled  Denominators are noted in all statistics and tables |  |
|  |  | **17** | Any analyses of variability in diagnostic accuracy, distinguishing pre-specified from exploratory  L211: We performed sensitivity analyses to examine the effect on assay performance of using only laboratory-confirmed survivor cases, and of removing controls found to have presence in an endemic area.  L317: Discordant results (section)  Thirteen survivor and 12 ‘unexposed’ control participants had DABA results discordant with their recruitment group.  Both the above were exploratory |  |
|  |  | **18** | Intended sample size and how it was determined  L169: Intended recruitment was 70 confirmed LF survivors and 70 unexposed controls to enable estimation of sensitivity and specificity with a precision of within +/- 10% based on the 95% confidence interval width for an observed estimate of 85%. Both study groups were a convenience sample of eligible individuals who were enrolled as they presented until the sample size was met |  |
|  | **RESULTS** |  |  |  |
|  | *Participants* | **19** | Flow of participants, using a diagram  L 229: Flow chart of study population recruitment and assay results |  |
|  |  | **20** | Baseline demographic and clinical characteristics of participants  L 224 244 : extensive text plus tables; Supporting Information 1 Table 1 |  |
|  |  | **21a** | Distribution of severity of disease in those with the target condition  Supporting Information 1 Table 1 : less relevant as test detects past infection and clinical information from original diagnosis is self reported. |  |
|  |  | **21b** | Distribution of alternative diagnoses in those without the target condition  Not applicable |  |
|  |  | **22** | Time interval and any clinical interventions between index test and reference standard  L302: Antibody reactivity in both plasma and oral fluid was slightly negatively correlated (Coef. -0.23 p 0.38, -0.35, p 0.07 respectively) with time elapsed since participants were ill (Figure 6), becoming significant when unreactive samples were excluded (Coef. -0.41 plasma p 0.001; -0.18 oral fluid p 0.03). |  |
|  | *Test results* | **23** | Cross tabulation of the index test results (or their distribution)  by the results of the reference standard  L281 Table 3: Sensitivity and Specificity of the LASV DABA test in plasma and oral fluid |  |
|  |  | **24** | Estimates of diagnostic accuracy and their precision (such as 95% confidence intervals)  L275: under field conditions the sensitivity and specificity of the LASV DABA in plasma was 81.7% (95% CI 70.7% - 89.9%) and 83.3% (72.7%- 91.1%) respectively and sensitivity and specificity of the LASV DABA in oral fluid was 71.8% (60.0 %– 81.9%) and 83.3% (72.7% - 91.1%) respectively.  Also L 339-345 : sensitivity analysis with different cutoff |  |
|  |  | **25** | Any adverse events from performing the index test or the reference standard  n/a |  |
|  | **DISCUSSION** |  |  |  |
|  |  | **26** | Study limitations, including sources of potential bias, statistical uncertainty, and generalisability  L363: Measuring the performance of the DABA assay was, however, affected by some important limitations of the study environment including the need to rely on original diagnostic tests and medical records to identify LF survivors, the lack of a field-feasible benchmark test for past infection, and the difficulty of obtaining truly unexposed controls in Sierra Leone……… (5 paragraphs of explanation and discussion ) |  |
|  |  | **27** | Implications for practice, including the intended use and clinical role of the index test  L346 Our findings, particularly those using oral fluid sampling, have important implications for the epidemiological research needed to clarify the burden of Lassa Fever infection and facilitate the development of candidate vaccines where understanding population serostatus is a critical issue.^25,26^  The ability to sample for a viral haemorrhagic fever without invasive blood-draw, thereby reducing risk of community reluctance and biosecurity collection hazards, opens up the possibility of performing the largescale seroprevalence surveys needed to clarify the geographical reach, range of severity, and transmission dynamics of LASV, not only in countries where there is known human incidence of disease but also in countries where the virus is endemic in rodent hosts.^27^ |  |
|  | **OTHER INFORMATION** |  |  |  |
|  |  | **28** | Registration number and name of registry  n/a |  |
|  |  | Supporting Information | Supporting Information |  |
|  |  | **30** | Sources of funding and other support; role of funders  L45: was funded by UK aid from the Department of Health and Social Care (<https://www.gov.uk/government/collections/official-development-assistance-oda--2>) via the UK Public Health Rapid Support Team Research Programme (Grant No. RST3_03). The funder had no role in study design, data collection and analysis, decision to publish or preparation of the manuscript. |  |
|  |  |  |  |  |

STARD 2015

### AIM

STARD stands for “Standards for Reporting Diagnostic accuracy studies”. This list of items was developed to contribute to the completeness and transparency of reporting of diagnostic accuracy studies. Authors can use the list to write informative study reports. Editors and peer-reviewers can use it to evaluate whether the information has been included in manuscripts submitted for publication.

### Explanation

A **diagnostic accuracy study** evaluates the ability of one or more medical tests to correctly classify study participants as having a **target condition.** This can be a disease, a disease stage, response or benefit from therapy, or an event or condition in the future. A medical test can be an imaging procedure, a laboratory test, elements from history and physical examination, a combination of these, or any other method for collecting information about the current health status of a patient.

The test whose accuracy is evaluated is called **index test.** A study can evaluate the accuracy of one or more index tests. Evaluating the ability of a medical test to correctly classify patients is typically done by comparing the distribution of the index test results with those of the **reference standard**. The reference standard is the best available method for establishing the presence or absence of the target condition. An accuracy study can rely on one or more reference standards.

If test results are categorized as either positive or negative, the cross tabulation of the index test results against those of the reference standard can be used to estimate the **sensitivity** of the index test (the proportion of participants *with* the target condition who have a positive index test), and its **specificity** (the proportion *without* the target condition who have a negative index test). From this cross tabulation (sometimes referred to as the contingency or “2x2” table), several other accuracy statistics can be estimated, such as the positive and negative **predictive values** of the test. Confidence intervals around estimates of accuracy can then be calculated to quantify the statistical **precision** of the measurements.

If the index test results can take more than two values, categorization of test results as positive or negative requires a **test positivity cut-off**. When multiple such cut-offs can be defined, authors can report a receiver operating characteristic (ROC) curve which graphically represents the combination of sensitivity and specificity for each possible test positivity cut-off. The **area under the ROC curve** informs in a single numerical value about the overall diagnostic accuracy of the index test.

The **intended use** of a medical test can be diagnosis, screening, staging, monitoring, surveillance, prediction or prognosis. The **clinical role** of a test explains its position relative to existing tests in the clinical pathway. A replacement test, for example, replaces an existing test. A triage test is used before an existing test; an add-on test is used after an existing test.

Besides diagnostic accuracy, several other outcomes and statistics may be relevant in the evaluation of medical tests. Medical tests can also be used to classify patients for purposes other than diagnosis, such as staging or prognosis. The STARD list was not explicitly developed for these other outcomes, statistics, and study types, although most STARD items would still apply.

### DEVELOPMENT

This STARD list was released in 2015. The 30 items were identified by an international expert group of methodologists, researchers, and editors. The guiding principle in the development of STARD was to select items that, when reported, would help readers to judge the potential for bias in the study, to appraise the applicability of the study findings and the validity of conclusions and recommendations. The list represents an update of the first version, which was published in 2003.

More information can be found on [http://www.equator-network.org/reporting-guidelines/stard](http://www.equator-network.org/reporting-guidelines/stard/).
