# Supplementary material for: Boosting understanding of Lassa Fever virus epidemiology: Field testing a novel assay to identify past Lassa Fever virus infection in blood and oral fluids of survivors and unexposed controls in Sierra Leone
Source: PLoS Negl Trop Dis. 2021 Mar 31;15(3):e0009255. doi: 10.1371/journal.pntd.0009255 (PMC8041174; doi:10.1371/journal.pntd.0009255)
Supplement: S1 Text — A. In-country laboratory validation processes. B. DABA Total Antibody Assay Information for Users. (DOCX) [file pntd.0009255.s002.docx]

S1_Text: Laboratory Appendix

- 1. Field laboratory processes
  2. DABA Total Antibody Assay Information for Users

**A. Field laboratory processes**

**DABA Total Antibody Assay**

The DABA Total Antibody Assay uses microwell strips coated with LASV Glycoprotein 2 (GP2) antigen (Native Antigen Company, UK), and a horseradish peroxidase (HRP) GP2 antigen (GP2 Sheep Fc-Tag-HRP) (Native Antigen Company, UK) conjugate (MicroImmune, Clin Tech, UK) to detect all antibodies that specifically bind LASV GP2 antigen. For the DABA ELISA, plasma samples were diluted 1:5 in serum diluent (MicroImmune, Clin Tech, UK). Oral fluid samples were used without further dilution following initial preparation in TM. Diluted positive controls and study samples were transferred to the coated microwells (100 µL/well) and incubated for 60 minutes at 37°C. Microwells were washed five times with 300 µL/well of wash buffer (MicroImmune, Clin Tech, UK) using an automated ELISA plate washer. HRP conjugated LASV GP2 was added to the microwells (100 µL/well) and incubated at 37°C for 120 minutes. Microwells were again washed five times with 300 µL/well wash buffer. TMB substrate (MicroImmune, Clin Tech, UK) was added (100 µL/well) and incubated for 30 minutes followed by stop solution (MicroImmune, Clin Tech, UK) (50 µL/well). Microwells were read at 450 nm with an OD450.

**IgG GP2 ELISA capture assay**

IgG GP2 ELISA capture assay uses microwell strips coated with rabbit anti-human IgG (AffiniPure™) at 5 µg/ml and a horseradish peroxidase (HRP) GP2 antigen (GP2 Sheep Fc-Tag-HRP) (Native Antigen Company, UK) conjugate (MicroImmune, Clin Tech, UK) to detect IgG antibodies that specifically bind LASV GP2 antigen. For IgG ELISA, plasma from study participants was diluted 1:200 and 1:50 in TM, oral fluid samples were used without further dilution following initial preparation. Diluted positive controls, and test samples (plasma and oral fluid) were transferred to microwells (100 µL/well) and incubated for 60 minutes at 37°C. Microwells were then washed five times with 300 µL/well wash buffer using an automated ELISA plate washer. HRP conjugated LASV GP2 was added to the microwells (100 µL/well) and incubated at 37°C for 30 minutes. Microwells were again washed five times with 300 µL/well wash buffer. TMB substrate was added (100 µL/well) and incubated for 30 minutes followed by stop solution (50 µL/well). Microwells were read at 450 nm with an OD450.

**DABA Quantification Assay:**

Six pilot plasma specimens from convalescent Sierra Leonean LF survivors with the highest end point reactivity dilutions were pooled (5ul of each sample taken to give total of 30ul). The pool was diluted further 1:10 in NHP and assigned as 1000 Arbitrary Units/ml (AU/ml). A set of 12x Quantification Standards were prepared from doubling dilutions in NHP to give a range of 1000 AU/ml to 0.49 AU/ml. Following testing of the 12x Standards and evaluation of the raw ODs obtained, it was determined that 9x Standards (covering a range of 500 AU/ml to 1.95 AU/ml) produced the ‘best-fit’ coverage at both top and bottom end of a Standard Curve.

Due to the small volume of plasma specimens available, there were insufficient volumes for use in DABA Quantification. A convalescent plasma sample (CP), used as ‘Positive Control’ material, was diluted 1:50 in NHP and doubling dilutions prepared as an alternative. The 1:50 dilution was matched to the pilot plasma specimen Standard Curve to determine an assigned arbitrary unit (AU/ml). The 1:50 CP dilution was assigned as 260 AU/ml; the remaining eight doubling dilutions were assigned as follows (130 AU/ml to 1.02 AU/ml). There was a good correlation between both sets of Standards in determining an AU. For standard curves, 2ul sample was added to 98ul serum diluent, then multiplied by 10 to determine AU/ml.

**B. Information for Users (IFU)**

**Double Antigen Binding Assay (DABA) for the Detection of Antibody to Lassa Virus GP2 Glycoprotein: Information for Users**

**DRAFT: 11 November 2019**

**FOR RESEARCH PURPOSES ONLY – not for diagnostic use**

1. **SUMMARY**

Lassa Double Antigen Bridging Assay (DABA) has been developed to detect antibodies to the Lassa Virus Glycoprotein 2 (GP2), corresponding to amino acids 259-426 of the Lassa virus glycoprotein precursor (Nigerian Strain GA391).

The Lassa DABA EIA is designed to be sensitive and specific using recombinant protein: recombinant Lassa Virus-GP2 Antigen (The Native Antigen Co.) coated solid phase and recombinant Lassa Virus-GP2 Antigen (The Native Antigen Co.) conjugated to Horse Radish Peroxidase (HRP).

1. **INTENDED USE**

This kit is a species neutral enzyme-linked immunosorbent assay (ELISA) for the qualitative detection of antibodies to Lassa virus GP2 in serum or plasma from humans and from other mammals, excluding camels. It is intended as a primary screening assay and for use as a second assay for /samples been found to be ‘screen reactive’ using an alternative Lassa antibody detection assay.

1. **PRINCIPLE OF THE TEST**

DABA Enzyme Linked Immunoassays are two-sequential-step ELISA that utilise a recombinant or purified antigen pre-coated onto a ‘solid phase’ (usually polystyrene microwell strips), which acts as to capture antibody in a first incubation. In a second incubation a second antigen of the same type, conjugated to tracer enzyme-horseradish peroxidase (HRP), is used as the detector for antibody captured in the first incubation. In the presence of Lassa GP2 antibodies, the pre-coated antigen specifically captures antibody from the plasma/serum.

After washing, conjugated antigen will bind to the free variable domains of the antibody during second incubation, thus a specific antigen-antibody-antigen-HRP immune-complex is developed on the solid phase. After the addition of tetramethylbenzidine (TMB) Substrate, the colourless chromogens are hydrolysed by the bound HRP conjugate to a blue coloured product. The blue colour turns yellow after stopping the reaction with 2M sulphuric acid. The presence of colour within the test well indicates the presence of antibodies to Lassa GP2 in the sample. The amount of colour can be measured and is proportional to the amount of antibody present. Wells containing samples negative for Lassa GP2 antibody remain colourless.

1. **REAGENTS**

Each kit contains sufficient materials for 96 tests. The shelf life of each kit is as indicated on the label fixed to the box containing the kit. All components must be stored at 2-8^o^C unless otherwise stated.

Materials Provided:

| **Coated Wells**: | One plate of 96 wells coated with recombinant Lassa GP2 antigen. Allow the wells to reach room temperature (18 to 30°C) before removal from the bag. Place unused wells in the sealable storage bag provided and return to 2-8°C. |
| --- | --- |
| **Negative Control**: | One bottle containing 200 µl of Negative Control. Ready to Use. |
| **Positive Control** | One bottle containing 100 µl of Positive Control (convalescent patient plasma). Ready to Use. |
| **Sample Diluent:** | One bottle containing 10 ml of Sample Diluent. Ready to Use |
| **Conjugate Diluent**: | One bottle containing 10 ml of phosphate buffered saline, protein stabiliser, inactivated HRPO and detergent. Ready to Use. |
| **Conjugate Concentrate:** | One bottle containing 100 µl of stock 100 times concentrated strength recombinant Lassa GP2 antigen coupled with horseradish peroxidase in stabiliser solution. **NB: Vortex gently then centrifuge the tube to ensure the full volume of Conjugate Concentrate is in bottom of tube prior to opening.**  Add one volume of Conjugate Concentrate to 99 volumes (ie 1:100 dilution) of required Conjugate Diluent to get the required Working Strength Conjugate Solution. Use within 8 hours of preparation. |
| **TMB Substrate - Ready to Use:** | One bottle containing 10 ml of 3,3’,5,5’- tetramethylbenzidine and stabilisers in a colourless solution (TMB Substrate). Ready to Use.  Keep Substrate Solution away from sunlight. The Substrate Solution should be colourless; if it is purple before being used, it should be discarded, and fresh Substrate Solution used.  Once opened, the bottle of TMB Substrate is stable refrigerated (2-8°C) for 30 days but must be discarded if crystals have formed. |
| **Stop Solution (2M H_2_SO_4_)**: | One bottle containing 5 ml of 2M Sulphuric Acid (H_2_SO_4_). Ready to Use. |
| **Wash Fluid**: | One bottle containing 100 ml of 10 times concentrated strength Wash Buffer Solution.  Add one volume of Wash Fluid Concentrate to 9 volumes of distilled or deionised water to give the required volume or dilute the entire contents of one bottle of Wash Fluid to a final volume of 1000 ml.  Store the working strength Wash Fluid at 18-30°C in a closed vessel under which conditions it will retain activity for one month. |

Additional Material and Instruments Required But Not Provided:

Good quality deionised or distilled water

Clean vessels for wash solution preparation

Micro-titre plate cover

Micropipettes and disposable tips capable of delivering 200 µL, 100 µL, 20 µL and 1-5µl volumes.

Waste discard container with disinfectant

EIA plate reader capable of reading optical density at 450nm (and 620-650nm).

Incubator, 37°C

**5. SPECIMEN COLLECTION AND PREPARTION**

**Handle all blood, serum and plasma as potentially infectious material.**

Serum and plasma (EDTA, citrated or heparinised) samples are suitable specimens for the test and should be obtained from whole blood using standard laboratory procedure.

Either fresh or thawed serum or plasma samples can be used for this assay. If not used immediately, they can be stored at 2-8°C for one week. Care should be taken to ensure that the serum samples are clear and not contaminated by microorganisms. Plasma samples collected into EDTA, sodium citrate or heparin may be tested, but highly lipaemic, icteric or haemolysed samples should not be used as they can give false results in the assay. Do not heat inactive samples.

1. **STORAGE AND STABILITY**

When stored at 2-8°C, the kit is stable up to the expiration date printed on the kit label.

1. **PRECAUTIONS AND SAFETY**

The serum in which the Positive pool (donors sero-negative for Lassa antigen prior to sampling) has been diluted to give the Positive Control and Negative Control serum are not reactive for antibodies to HIV 1 and 2, HCV and for Hepatitis B surface antigen. The controls should be handled and disposed of as though potentially infectious.

The TMB substrate solution contains 3, 3', 5, 5' tetramethylbenzidine and has been reported to be non-carcinogenic. Avoid direct skin- contact with the reagent. Wear latex gloves when handling this reagent. If TMB comes into contact with the skin, wash immediately with water.

The Stop Solution contains sulphuric acid (2M). Contact with skin and mucous membranes should be avoided. If the Stop Solution comes into contact with these sites, rinse with copious amounts of water.

Wear disposable gloves when handling clinical specimens and kit components. Treat all clinical specimens and controls and any materials coming into contact with them as potentially infectious.

Dispose clinical material and potentially infected materials in accordance with local regulations.

Avoid microbial contamination of reagents. Do not use reagents that show signs of contamination.

Good laboratory procedure should be employed to avoid cross contamination of samples and reagents. Take out only the required volume of reagent from the original container (usually 0.9-1.0 mL per strip) for dispensing into wells. Discard unused reagents - do not return unused reagent to original containers.

**8. ASSAY PROCEDURE**

**Step 1: Reagents preparation**

Allow all reagents to reach room temperature (18-30°C) prior to use. This is VERY IMPORTANT.

Check the Wash Buffer concentrate for the presence of salt crystals. If crystals have formed in the solution, re-suspend by warming at 37°C until the crystals have dissolved. Dilute the stock bottle concentrated wash fluid buffer 10x times with distilled or deionised water. Use a clean vessel to dilute the buffer. It is recommended that working strength Wash Buffer be prepared as required on the day of use. Remaining Wash Buffer Concentrate stock should be re-stored at 2-8°C if not used.

Prepare the ‘Working Strength’ Conjugate Solution (see Materials Provided section for preparation details). Once prepared ‘Working Strength’ Conjugate Solution should be used within 8 hours.

If testing a total of 96 samples (full plate) – dispense 9.9ml of Conjugate Diluent into a separate vessel and then add 100µl of the Conjugate Concentrate provided, to give ‘Working Strength’ Conjugate reagent. Mix well before use.

(Alternatively, for a total of 96 tests (full plate) – 100µl of Conjugate Diluent can be removed from the Conjugate Diluent bottle (containing 10ml), and this volume replaced with the full amount (100µl) of the Conjugate Concentrate provided, to give ‘Working Strength’ Conjugate reagent. Mix well before use).

If testing a total of 48 samples (1/2 plate) – dispense 4.95ml of Conjugate Diluent into a separate vessel and then add 50µl of the Conjugate Concentrate provided, to give ‘Working Strength’ Conjugate reagent. Mix well before use.

If testing a total of 24 samples (1/4 plate) – dispense 2.475ml of Conjugate Diluent into a separate vessel and then add 25µl of the Conjugate Concentrate provided, to give ‘Working Strength’ Conjugate reagent. Mix well before use.

All other reagents are provided Ready-to-Use.

**Step 2: Numbering wells**

Remove and assemble the required number of recombinant Lassa GP2 antigen-coated microwell strips to perform the test. A minimum of 6 wells is needed for the controls (2x Positive Controls and 4x Negative Controls) which must be included in each test run. Set the strips needed into strip-holder and only use the required number of strips for the test. (Use the Lassa GP2 DABA ELISA Frontsheet Template to record the sample identity numbers to be tested and procedural information provided at the end of this IFU).

**Step 3: Adding controls and samples**

Following a DABA ELISA Frontsheet Template as a guide, **pipette** **80µl of Sample Diluent** **and 20µl of the Positive Control** into their respective well positions (1A – 1B)**. Pipette 80µl of Sample Diluent and 20µl of Negative Control** into their respective well positions (1C – 1F). **Pipette 80µl of Sample Diluent and 20µl of each Sample** **into designated well positions** (1G onwards). NB: only test the number of samples in a single test run that can be dispensed into assigned wells within ten minutes**.**

Note: use a separate disposal pipette tip for each Sample, Negative Control and Positive Control to avoid cross-contamination.

**Step 4: Incubation**

Cover the plate with a plate sealer and mix gently by tapping the side of the plate strip holder. Incubate **at 37 ± 2°C in a moist chamber or dry incubator for 60 ± 2 minutes** (If a dry incubator is used do not open the door frequently).

**Step 5: Washing**

After the end of the incubation, remove and discard the plate sealer. Wash each well **5 times** with diluted Wash Buffer (see reagent preparation). The wash cycle is carried out as follows: aspirate the contents of the well and dispense at least 300 µl per well of Wash Buffer to form a meniscus. Allow the microwells to soak for 30-60 seconds and then aspirate. Repeat the wash cycle a further four times. Alternatively, an automatic plate washer may be used. After the final washing cycle turn down the strips plate onto blotting paper or clean towel and tap the plate to remove any remaining Wash Buffer.

**Step 6: Conjugate**

**Dispense 100µl of ‘Working Strength’ Conjugate** into each well. This is best performed using a multi-channel pipette.

Cover the plate with a plate sealer and incubate **at 37 ± 2°C in a moist chamber or dry incubator for 120 ± 2 minutes** (If a dry incubator is used do not open the door frequently).

**Step 7: Washing**

After the end of the incubation, remove and discard the plate sealer. **Wash each well** **5 times** as in Step 5. After the final washing cycle turn down the strips plate onto blotting paper or clean towel and tap the plate to remove any remaining Wash Buffer.

**Step 8: Substrate**

Dispense **100µl of Ready to Use TMB Substrate** into each well. This is best performed using a multi-channel pipette.

Cover the plate with a plate sealer and mix gently by tapping the side of the plate strip holder. Incubate the plate at **37 ± 2°C in a moist chamber or dry incubator for 30 ± 2 minutes**, **avoiding light.** (If a dry incubator is used do not open the door frequently).

The enzymatic reaction between the substrate and conjugate will produce a blue colour in the positive control and any anti-Lassa GP2 positive sample wells.

**Step 9: Stopping reaction**

After the end of the incubation, remove and discard the plate sealer. Add **50µl of Stop Solution** into each well. This is best performed using a multi-channel pipette and the stop solution should be added using the same timing and sequence that was used to add the substrate solution. Mix gently by tapping the side of the plate strip holder. Following addition of Stop Solution, an intensive yellow colour will develop in the Positive Control and any anti-Lassa GP2 positive sample wells.

**Step 10: Measure the absorbance**

Calibrate the plate reader with the blank well and read the absorbance at 450nm (if a dual filter instrument is used, set the reference wavelength at 630 or between 620 and 650nm on the spectrophotometric plate reader). Calculate the cut-off value and evaluate the results.

**Note: read the absorbance within 10 minutes after stopping the reaction.**

**9. INTERPRETATION OF RESULTS AND QUALITY CONTROL**

Each microplate must be considered separately when calculating and interpreting results of the assay, regardless of the number of plates concurrently processed. The results are calculated by relating each sample optical density (OD) value to the cut-off value (CO) of the plate.

Calculation of cut-off value for :

Cut-off value (CO) = NC_mean_ + 0.1

NC_mean_ = the mean absorbance value for 4 Negative Controls

If one of the Negative Control values does not meet the Quality Control range specifications (see below), it should be discarded and the mean value calculated again using the remaining values.

Quality Control Range

Each sample absorbance test OD result (S) is valid if the Quality Control criteria are verified as below:

The absorbance value OD of each Negative Control must be less than 0.100

The absorbance value OD of each Positive Control must be greater than 1.000

Interpretation of results

Negative results (S/CO ≤1): Samples giving an absorbance less or equal to the cut-off value are considered negative, that is, no GP2 antibodies to Lassa Virus have been detected using this ELISA kit.

Positive results (S/CO >1): Samples giving absorbance greater than the cut-off value are positive for this assay, that is, GP2 antibodies to Lassa Virus have been detected with this ELISA kit.

To interpret results across plates, optical densities should be normalised using the mean of the plate’s negative controls + 0.1 / the sample result to produce a ratio described as a ‘normalised OD’

.

| **Annex 1: Lassa GP2 DABA ELISA - Frontsheet Template** | | | | | | | | | | | | | | | | | | | | | | | | | | | | | | | | | | | | | | | |  |  |
| --- | --- | --- | --- | --- | --- | --- | --- | --- | --- | --- | --- | --- | --- | --- | --- | --- | --- | --- | --- | --- | --- | --- | --- | --- | --- | --- | --- | --- | --- | --- | --- | --- | --- | --- | --- | --- | --- | --- | --- | --- | --- |
|  | **Kit Lot no:** | | |  | | | |  | **Plate Lot no:** | | |  | | | | | | |  | | | | **Operator:** | | | | |  | | | | | | | | | | | | | |
|  | **Expiry Date:** | | |  | | | |  | **Expiry Date:** | | |  | | | | | | |  | | | | **Test Date:** | | | | |  | | | | | | | | | | | | | |
|  |  | | |  |  | |  | | | |  | | | | |  | | | |  | | | | |  | | | | | | | | | |  | |  |  |  |  |  |
|  |  | | |  | PIPETTE DETAILS | | | | | |  | | |  | | | |  | | | | | | |  | | | |  | | |  |  | | | | | |  |  |  |
|  | **MULTICHANNEL 50-300 µl** | | | |  | | | | | |  | | |  | | |  | | | | | | | MODEL | | | | | | INVENTORY No | | | | | | | | | | | |
|  | **SINGLE 200 µl** | | | |  | | | | | | INCUBATOR | | | | | | TEMPERATURE | | | | | | |  | | | | | | Serial No: ________ | | | | | | | | | | | |
|  | **SINGLE 100 µl** | | | |  | | | | | | WASHER | | | | | |  | | | | | | |  | | | | | | Serial No: ________ | | | | | | | | | | | |
|  | **SINGLE 20 µl** | | | |  | | | | | | READER | | | | | | 450/630 nm | | | | | | |  | | | | | | Serial No: ________ | | | | | | | | | | | |
|  | **SINGLE 1-5 µl** | | | |  | | | | | |  | | |  | | | |  | | | | | | |  | | | |  | | |  |  | | | | | |  |  |  |
|  |  | | |  |  | |  | | | |  | | | | |  | | | |  | | | | |  | | | | | | | | | |  | |  |  |  |  |  |
| **Plate Layout** | | | |  |  | |  | | | |  | | | | | |  | | | |  | | | | |  | | | | | | | | | |  | |  |  |  |  |
|  | | **1** | **2** | | | **3** | **4** | | | **5** | | | **6** | | **7** | | | | **8** | | | **9** | | | | | **10** | | | | **11** | | | **12** | | | | | | |  |
| A | | POS CTL |  | | |  |  | | |  | | |  | |  | | | |  | | |  | | | | |  | | | |  | | |  | | | | | | |  |
| B | | POS CTL |  | | |  |  | | |  | | |  | |  | | | |  | | |  | | | | |  | | | |  | | |  | | | | | | |  |
| C | | NEG CTL |  | | |  |  | | |  | | |  | |  | | | |  | | |  | | | | |  | | | |  | | |  | | | | | | |  |
| D | | NEG CTL |  | | |  |  | | |  | | |  | |  | | | |  | | |  | | | | |  | | | |  | | |  | | | | | | |  |
| E | | NEG CTL |  | | |  |  | | |  | | |  | |  | | | |  | | |  | | | | |  | | | |  | | |  | | | | | | |  |
| F | | NEG CTL |  | | |  |  | | |  | | |  | |  | | | |  | | |  | | | | |  | | | |  | | |  | | | | | | |  |
| G | |  |  | | |  |  | | |  | | |  | |  | | | |  | | |  | | | | |  | | | |  | | |  | | | | | | |  |
| H | |  |  | | |  |  | | |  | | |  | |  | | | |  | | |  | | | | |  | | | |  | | |  | | | | | | |  |
|  | |  |  | | |  |  | | |  | | |  | |  | | | |  | | |  | | | | |  | | | |  | | |  | | | | | | |  |
| **OD Value read at 450/630 nm** | | | | | | | | | | | | | | | | | | | | | | | | | | | | | | | | | | | | | | | | |  |
|  | | **1** | **2** | | | **3** | **4** | | | **5** | | | **6** | | **7** | | | | **8** | | | **9** | | | | | **10** | | | | **11** | | | **12** | | | | | | |  |
| A | |  |  | | |  |  | | |  | | |  | |  | | | |  | | |  | | | | |  | | | |  | | |  | | | | | | |  |
| B | |  |  | | |  |  | | |  | | |  | |  | | | |  | | |  | | | | |  | | | |  | | |  | | | | | | |  |
| C | |  |  | | |  |  | | |  | | |  | |  | | | |  | | |  | | | | |  | | | |  | | |  | | | | | | |  |
| D | |  |  | | |  |  | | |  | | |  | |  | | | |  | | |  | | | | |  | | | |  | | |  | | | | | | |  |
| E | |  |  | | |  |  | | |  | | |  | |  | | | |  | | |  | | | | |  | | | |  | | |  | | | | | | |  |
| F | |  |  | | |  |  | | |  | | |  | |  | | | |  | | |  | | | | |  | | | |  | | |  | | | | | | |  |
| G | |  |  | | |  |  | | |  | | |  | |  | | | |  | | |  | | | | |  | | | |  | | |  | | | | | | |  |
| H | |  |  | | |  |  | | |  | | |  | |  | | | |  | | |  | | | | |  | | | |  | | |  | | | | | | |  |
