# Supplementary material for: Boosting understanding of Lassa Fever virus epidemiology: Field testing a novel assay to identify past Lassa Fever virus infection in blood and oral fluids of survivors and unexposed controls in Sierra Leone
Source: PLoS Negl Trop Dis. 2021 Mar 31;15(3):e0009255. doi: 10.1371/journal.pntd.0009255 (PMC8041174; doi:10.1371/journal.pntd.0009255)
Supplement: S2 Text — A. Protocol. B. Consent/Assent form. C. Questionnaires and control eligibility check list. D. Patient information leaflet. (DOCX) [file pntd.0009255.s003.docx]

**S2_Text Study Tools**

- 1. Protocol
  2. Consent/assent form
  3. Questionnaires and control eligibility check list
  4. Patient information leaflet

1. **Study protocol**

UK PUBLIC HEALTH RAPID SUPPORT TEAM RESEARCH

**Field Validation Study of an ELISA assay to identify**

**Lassa virus specific antibody responses in oral fluids**

**Case-control assessment of a novel immunoassay comparing antibody responses from oral fluids and blood samples in Lassa Fever survivors and unexposed controls in Sierra Leone**

**Study protocol**

**Version**: 31 January 2019

**Principal investigators**

Dr Donald S. Grant

Physician-in-Charge: National Lassa Fever Unit, Kenema Government Hospital, Sierra Leone

District Medical Officer, Kenema

Email: [donkumfel@yahoo.co.uk](mailto:donkumfel@yahoo.co.uk)

Ms Hilary Bower

UK Public Health Rapid Support Team, London School of Hygiene and Tropical Medicine

Email [Hilary.bower@lshtm.ac.uk](mailto:Hilary.bower@lshtm.ac.uk)

Professor Richard S. Tedder

Imperial College, London

Email: [richardtedder123@gmail.com](mailto:richardtedder123@gmail.com)

**Co-investigators**

Dr John S. Schieffelin

Assistant Professor of Pediatrics and Internal Medicine, Tulane University School of Medicine

Email: [jschieff@tulane.edu](mailto:jschieff@tulane.edu)

Dr Benedict Gannon

UK Public Health Rapid Support Team, Public Health England

Email: [ben.gannon@phe.gov.uk](mailto:ben.gannon@phe.gov.uk)

Dr Onome Akpogheneta

Dept of Infectious Disease Epidemiology, London School of Hygiene and Tropical Medicine

Email [onome.akpogheneta@lshtm.ac.uk](mailto:onome.akpogheneta@lshtm.ac.uk)

Contents

[1 Summary 3](#_Toc536626223)

[2 Introduction 4](#_Toc536626224)

[2.1 Host & transmission 5](#_Toc536626225)

[2.2 Sero-prevalence & incidence of infection 5](#_Toc536626226)

[3 Aims and objectives 6](#_Toc536626227)

[4 Methods 7](#_Toc536626228)

[4.1 Recruitment 7](#_Toc536626229)

[4.2 Study participants 8](#_Toc536626230)

[4.4 Eligibility & consent 8](#_Toc536626231)

[4.5 Sample collection 9](#_Toc536626232)

[4.6 Data collection 9](#_Toc536626233)

[4.7 Sample size 9](#_Toc536626234)

[4.8 Data analysis 10](#_Toc536626235)

[4.9 Dissemination of results 10](#_Toc536626236)

[5 Human subjects’ protection 11](#_Toc536626237)

[5.1 Risks to participants 11](#_Toc536626238)

[5.2 Intellectual property 12](#_Toc536626239)

[5.3 Data handling 12](#_Toc536626240)

[5.4 Storage of study participants’ samples 12](#_Toc536626241)

[6 References 13](#_Toc536626242)

[7 Appendices 14](#_Toc536626243)

[7.1 Consent/assent form 14](#_Toc536626244)

[7.2 Questionnaires and control eligibility check list 14](#_Toc536626245)

[7.3 Patient information leaflet 14](#_Toc536626246)

# Summary

This proposal describes a collaborative study between the UK Public Health Rapid Support Team (UK-PHRST), London School of Hygiene and Tropical Medicine (LSHTM), and Kenema Government Hospital (KGH), Sierra Leone to develop and validate a novel enzyme-linked immunosorbent assay (ELISA) to investigate antibody responses to Lassa virus (LASV) infection in oral fluid samples.

The study will compare oral fluid and blood samples from Lassa Fever (LF) survivors and unexposed controls in order to validate oral fluid samples as a means of establishing anti-LASV antibody prevalence in endemic settings.

There is limited recent evidence on the prevalence of LASV infection and the longevity of immune responses to LASV within endemic communities. A robust antibody assay suitable for use with non-invasive oral fluid sampling could greatly improve ability to accurately estimate prevalence of infection, describe the spectrum of disease, and facilitate longitudinal incidence, immunity and transmission studies through improved acceptance of testing.

A prototype ELISA for use with oral fluid has been created in the UK by experts in this form of testing. A small number of stored convalescent human samples (n=6) from Sierra Leone consented for research purposes will be obtained through official channels to finalise the prototype development in the UK. Validation of the assay, including determination of sensitivity and specificity, will be done in Sierra Leone by national laboratory staff supported by UK-PHRST laboratory scientists, using oral fluids and blood samples collected from individuals confirmed to have had LF by the Lassa Fever Unit of KGH, Kenema, Sierra Leone. Samples from individuals without LF diagnosis or known LASV exposure will be collected as controls.

A validated non-invasive, community-acceptable, and logistically simpler tool for measuring LASV sero-prevalence and sero-conversion in communities in endemic countries will greatly facilitate the understanding of LASV epidemiology and support the development and trialling of LASV vaccines and other control measures.

# Introduction

Lassa Fever (LF) is a viral haemorrhagic illness endemic in parts of West Africa including Sierra Leone, Liberia, Guinea, Mali and Nigeria where the illness was first identified.^1^ In endemic settings, it is believed that the majority of those infected with Lassa virus (LASV) experience sub-clinical infection or mild disease with only around 20% infections resulting in hospitalisation.^2^ Overall mortality from infection is thought to be ~1% but in hospitalised cases, case fatality rates of up to 50 to 70% have been reported,^3^ often linked to late presentation. LF outbreaks can cause significant public health emergencies, often triggered by patients presenting in health facilities that are unaccustomed to receiving haemorrhagic fever cases, with the accompanying risks of health worker and nosocomial infection, as well as the burden of investigating and managing large numbers of suspect cases.

During clinical LASV infection, early symptoms may include fever, headache, and muscle aches, mimicking symptoms of diseases such as malaria and typhoid fever which are common in LASV endemic areas. As clinical infection progresses, patients may go into shock, develop multi-organ failure, and sometimes bleed from the mouth, nose, eyes, or rectum.^4^ In addition, there is evidence that LF survivors suffer long-term sequelae such as hearing loss, depression, joint pain and hair loss.^3^ There are currently no vaccines to prevent LF nor approved drugs to treat, and although accepted wisdom is that early clinical use of the nucleoside drug ribavirin can reduce mortality in clinical cases, there is little scientific evidence to support this.

It is estimated that up to 300,000 LASV infections occur annually resulting in 5,000 deaths,^2^ but these data are based largely on the results of a single study carried out in the 1980s.^5^ Similarly, there is little evidence for the 20-80% proportions cited for sub-clinical and clinical disease. The need for more robust estimates based on up-to-date data has been recently emphasised by WHO in its Research & Development Blueprint which identified LASV as one of eight priority diseases for urgent attention.^6^ More precise estimates of these parameters, and deeper understanding of the exposure and immune status of populations in the endemic countries, will also be critical to developing, targeting, and evaluating the efficacy of LASV vaccine candidates and other control measures.

LASV is an arenavirus composed of an enveloped, segmented negative single-stranded RNA and classified as a Category A pathogen requiring high biosafety handling.^7^ Several methods are used to diagnose LF cases including reverse transcriptase polymerase chain reaction (RT-PCT), a recombinant LASV (ReLASV) ELISA, as well as antigen detection tests and a lateral flow immunoassay rapid diagnostic test, all of which require blood samples.^8^ Virus isolation by cell culture is the gold standard for LASV diagnostic purposes, but cannot be performed in normal diagnostic facilities due to the high-level biosafety laboratory facilities required for live virus work.

## 2.1 Host & transmission

The natural host for LASV is the common African soft-furred rat, *Mastomys natalensis*, in which infection is thought to be lifelong and harmless. Recently there have been reports of other host rodents and investigations into this are continuing.^9^ Viral transmission occurs - via aerosol or contact - from infected rats or their excretions to humans, and subsequent LASV infection in humans can be lethal. The virus can also be transmitted between humans via blood and tissue fluids, particularly in hospitals with weak infection prevention control measures, and among contacts exposed to body fluids through caring practices, resulting in high mortality rates.^3, 10^

## 2.2 Sero-prevalence & incidence of infection

At present, sero-prevalence and persistence of LASV antibodies and incidence of infection (measured by sero-conversion) can only be studied through surveys using blood-based sampling. Significant bio-security, community and psycho-social issues accompany the collection of blood samples in affected countries. Indeed, the drawing of blood can be one of the most contentious issues in a clinical trial process potentially arousing political, cultural and social antipathy,^11-14^ as well as requiring considerable logistics to correctly and safely collect and store specimens.

“Oral fluid” is found in the gingival crevice (between teeth and gums) and contains considerably higher levels of IgG than saliva.^15^ Oral fluid sampling has major advantages over blood collection: it is non-invasive, more acceptable to subjects of all ages (due to absence of pain and low or no perceived risk of contamination), and easier to collect without the need for medically-trained personnel. It is safer for collectors, removing the risk of needle-stick injury and other collection and storage-related exposures.^16^

The availability of a non-blood-based, non-invasive alternative method to detect LASV antibodies, and thus past infection, would allow more comprehensive sampling due to its higher acceptability by populations. This would greatly facilitate the community-based research needed to gather critical information on the full burden of LASV infection and disease and on levels of immunity, while also potentially providing a non-invasive tool to track responses to immunisation when trials are underway.

Oral fluid has been used in surveillance activities to detect antibodies to viral infections such as mumps, measles, rubella, and is routinely used for the diagnosis of HIV and Hepatitis A and C.^15, 17^ During the 2013-16 West Africa Ebola outbreak, a reverse IgG-capture ELISA for oral fluid samples was developed by the Public Health England (PHE) team involved in this proposal, and used to investigate the sero-prevalence of Ebola virus infection in affected households and communities, with high sensitivity (97.4%, 95% CI, 92.5%-99.5%) and specificity (99.7%, 95% CI, 98.4%-99.7%).^18^

**2.3 Assay development**

Laboratory scientists from Public Health England and Imperial College are in the process of developing a prototype LASV immunoassay for use with oral fluid incorporating single or combined LASV antigens (glycoprotein 1 (GP1), GP2 or recombinant matrix protein (rMP)). To complete the development stage and provide a reliable assay for validation, a small number of anonymous convalescent human samples will be requested from the KGH LF unit under a Material Transfer Agreement. Once the assay kits are complete, all subsequent validation work will be done in Sierra Leone in order to support increased research capacity in country.

**2.4 Study sites**

Kenema District is one of the ‘hotspots’ of Lassa Fever transmission in Sierra Leone and the KGH LF Unit is the national centre for diagnosis, treatment and research. Together with the Irrua Specialist Teaching Hospital in Nigeria, KGH is one of only two facilities globally where LASV testing and LF patient admission is continuous, and there is a programme of survivor monitoring and follow-up, which will facilitate the recruitment of the convalescent LF survivors needed for this validation work.

The KGH laboratory, medical and outreach teams at KGH have extensive experience of diagnosing treating and collecting data on LF, supported over many years by partners including Tulane University, New Orleans, USA. KGH carries out clinical and diagnostic research including an ongoing 5-year study of sequelae and immunity in survivors of viral haemorrhagic fevers (referred to hereafter as the Survivors Study). This validation study will be embedded in the Survivors Study for efficiency and to ensure a coordinated approach to survivors. The study presented here shares a principal investigator (Dr Grant) with the Survivors Study and the study teams will collaborate on all activities.

# Aims and objectives

The aim of this study is:

- To develop and examine the validity of a new assay to measure evidence of LASV infection for use with oral fluids by comparing naturally acquired antibody responses to LASV in oral fluids and blood serum samples from LF survivors and unexposed controls.

The specific objectives are:

1. To complete the development of a novel ELISA to investigate naturally acquired antibody responses LASV infection in non-invasive oral fluid samples
2. To validate this tool and determine its sensitivity and specificity by measurement of LASV-specific antibodies in oral fluids and blood samples from LF survivors and unexposed controls
3. To evaluate the feasibility of oral fluid as an alternative sampling tool using these findings
4. In LF survivors, to correlate LASV-specific antibody responses with time since clinical infection, original clinical characteristics and potential re-exposure events.

# Methods

## 4.1 Recruitment

**Inclusion Criteria**

For LF survivor participants

- History of LF as documented by a positive anti-LASV Ag or IgM ELISA in the KGH diagnostic laboratory
- Age ≥6 years
- No fever at recruitment (Temperature below 37.5^o^C)
- Willingness to provide informed consent (or assent, if applicable)
- Willingness to undergo phlebotomy for blood samples and provide oral fluid samples

For unexposed control participants

- No history of LF clinical illness and/or LASV positive test,
- No history of residence, work or visiting in LF endemic zones
- No history of contact with a LF case
- Age ≥6 years
- No fever at recruitment (Temperature below 37.5^o^C)
- Willingness to provide informed consent (or assent, if applicable)
- Willingness to undergo phlebotomy for blood samples and provide oral fluid samples

## 4.2 Study participants

Up to 70 LF survivors admitted to the KGH Lassa Ward for treatment of confirmed LF and discharged up to 15 years prior to the start of this study will be recruited. The study team will work with LF programme outreach staff at KGH to identify LF survivors and implement the study protocol.

Unexposed controls will be recruited from among individuals understood to have no exposure to the *Mastomys natalensis* rodent or LF cases. After agreement with the relevant authorities, control participants will be sought from among medical and/or nursing students in Freetown (an area considered outside the LASV endemic zone) as a relatively easily-accessed cohort of individuals for whom taking part in a research study may be of interest. The study brings no inherent benefit to participants, however those involved in study for health care/public health careers may benefit from taking part in a well-conducted research study. We will also collaborate with college authorities to offer lectures on the conduct of research studies and on LF as one of Sierra Leone’s priority emerging disease issues. A questionnaire including past travel/residence/contact information will be used to exclude candidates with possible exposure (Appendix 2: Control eligibility check).

## 4.4 Eligibility & consent

After holding meetings with key community leaders and Lassa survivor communities about the aims of the study, individual survivors will be approached for recruitment. The purpose and objectives of the study will be explained verbally, and a patient information leaflet provided for those able to read (Appendix 3: Patient information leaflet). Study investigators will explain the role of the participant in the study and discuss potential risks of participation. Informed consent will be in English. If English is not the patient’s native language or they are unable to read, the patient will be informed about the study and related risks in his or her native language by a collaborating investigator fluent in that language who will read the consent form to the potential participant in the presence of a witness.

Potential participants will have the opportunity to raise any concerns, after which, written informed consent will be sought from all adult participants and from adult guardians of child participants (Appendix 1: Consent form). For survivors, consent will include approval to access clinical records of their LF admission and to information previously given to the Survivors Study. All participants will be asked to consent to storage of their oral fluid and blood samples for possible future research; samples from participants who do not agree to storage will be destroyed after the validation study.

Following provision of informed consent, clinical, demographic and risk factor information for the participant will be collected from medical records completed during admission and from the Survivor Study database. Participants will be asked to respond to a short questionnaire specific to the validation study focussing on possible antibody-boosting exposures (Appendix 2: Questionnaires). Data will be entered into a password-protected electronic database.

## Sample collection

A 5ml blood sample will be collected and stored in a cool box for daily transfer to the laboratory. Serum will be processed then stored at -20°C prior to testing antibody responses.

Oral fluid will be collected by the participant rubbing a small sponge swab firmly on the gums for 90 seconds, then placing it in a stoppered plastic tube and in a sealable plastic bag and stored in a cool box, then transferred for storage at -20°C at the end of the day prior to testing antibody responses. Experience suggests that oral fluid collection is acceptable to participants of all ages.^18^

Oral fluid and blood samples will be tested to detect anti-LASV antibodies using the new ELISA tool. Further comparative tests may be conducted with assays currently in use in KGH.

## Data collection

Data will be collected using a study-specific questionnaire (Appendix 2), participants’ medical records during admission to KGH Lassa Fever Unit, and data held in the Survivors Study database. Variables for survivors will include demographics, symptoms and clinical information at the time of admission, dates of LF onset and discharge, laboratory test results at discharge, and information on any known exposure to LASV or LF patients since recovery, in order to explore potential immunity-boosting events.

Unexposed participants will be asked a series of questions regarding exposure to LASV hosts and/or cases to determine if they fulfil the inclusion criteria, and if not excluded based on responses, will be asked to respond to a brief demographic questionnaire.

Both groups will be asked to give feedback on using the oral fluid swab, including perceptions of the method as a follow-up and research tool.

## Sample size

We will recruit at least 70 LF survivors and 70 unexposed controls. This will allow for an estimation of specificity of at least 61.2% at 95% confidence interval (CI) with10% relative precision if true sensitivity is 85%, and higher precision if true sensitivity is greater (Table 1).

**Table 1: Sample size estimation for a range of sensitivity levels**

| Sensitivity point estimate (%) | Relative precision (%) | | | |
| --- | --- | --- | --- | --- |
|  | 5 | 10 | 20 | 50 |
| 80 | 385 | 97 | 25 | 4 |
| 85 | 272 | 68 | 17 | 3 |
| 90 | 171 | 43 | 11 | 2 |
| 95 | 81 | 21 | 6 | 1 |

## Data analysis

Analyses will:

- Determine the sensitivity and specificity of the novel assay to detect naturally acquired LASV-specific antibodies in LF survivors and unexposed controls in oral fluids compared to blood samples
- Describe antibody levels among study participants as a function of time since clinical infection and in relation to original clinical characteristics and potential re-exposure events.
- Investigate characteristics of any apparently unexposed controls found to have positive antibodies

## Dissemination of results

The results of the study will be disseminated through reports, academic papers, and presentations. We will ensure that negative results are also reported. All publications will be open-access. We will also share the results directly with the LASV team at KGH, government of Sierra Leone and other organizations working on LASV. A report will be shared with the Sierra Leone Ethics and Scientific Review Committee.

Survivor participants will not be informed of their individual results as knowledge carries no individual benefit to health or wellbeing. Unexposed controls will be informed if they are found to have positive antibodies and the meaning of this will be explained.

# Human subjects’ protection

## Risks to participants

The study protocol will be submitted to the Sierra Leone Ethics and Scientific Review Committee and at LSHTM for approval. No implementation will begin until approval is received. Written informed consent will be sought as described in section 2.3 above.

There is a risk that participants may not be properly informed about the study. Risks of drawing blood from participants are typically minor, with a bruise at the puncture site being the most common adverse event and a very small risk of infection. There is risk that participants may experience duress during the recruitment and interview process or in providing samples for analysis and a risk of loss of privacy. There are no risks in providing oral fluids.

We will take appropriate measures to minimize these risks to participants by providing clear training, guidance and supervision to members of the study team. We will be sensitive to any breach of confidentiality due to the stigmatizing nature of LASV.^19^ Study data will be managed according to a clear operational protocol, with only authorised research team members having access to de-identified data. We will also minimise risk to participants by engaging staff who have already built a rapport with LF survivors.

There will be no costs to the participant for participating in this research study. All costs related to the study, including phlebotomy and laboratory testing, will be paid for by the study.

Recruitment and participation will require a maximum time commitment of 1 hour from each participant, financial compensation of 100,000 Leones (approximately $11) will be provided for inconvenience, transport and loss of work time. This amount is consistent with the amount paid per follow-up visit by the Survivors Study.

Child survivors will be recruited into the study after the informed consent from their parent or guardian. In accordance with Sierra Leonean age cut-offs for child participation in research, children older than 12 years will also be asked to provide assent to participate. Pregnant women will be eligible to participate in either study cohort.

Participants will be informed that participation is voluntary and that they are free to discontinue their participation at any time with no adverse consequence. As the primary aim of this work is to validate an assay, individuals who do not wish to provide any oral fluid and/or blood samples will not be recruited. Participation in, or discontinuation from the study will not affect the participant’s ability to receive medical care provided by the Sierra Leone health service. The study will not offer or provide any medical care, other than to respond to an emergency unexpected reaction to the sampling procedure.

## Intellectual property

Participants will be informed that the researchers and study team will not benefit financially from the development and testing of this assay. No intellectual property rights (IPR) will be sought for the new assay, and IPR linked to the conjugate component of the assay will be waived for production of the assay by state laboratories in endemic countries. Sample donors will be asked to freely donate a single blood and oral fluid sample and to relinquish all rights, title and interest to these samples.

## Data handling

Maintaining participant confidentiality is an essential part of medical research. Staff will receive training on ensuring patient confidentiality, and all data collected on study participants will be secured (locked if paper or password-protected/encrypted if electronic). All participants will be assigned a unique identifying number. An electronic file as well as a paper log book will be kept by the study coordinator linking patient names with the unique identifying number. All electronic master lists will be kept on password-protected computers in locked offices or in the restricted access laboratory in the Lassa unit. Paper log books will be kept in a locked cabinet in locked offices. All electronic copies of medical records and laboratory documents will be kept on password-protected computers in locked offices with restricted access.

An anonymised version of the raw database(s) stripped of all identifying information such as participant name, address, etc. will be created for analysis. Only authorised members of the LSHTM and Lassa Fever Unit data team will have access to the raw data including the personal identifiers, and only for the purpose of entering the information into an electronic database, checking for errors, and producing the anonymised version of the database for analysis at LSHTM. The electronic database will be password-protected and stored on a secure server owned by the London School of Hygiene & Tropical Medicine.

De-identified data will be shared with KGH investigators. No individual data or samples will be sold to or shared with third parties beyond the collaboration.

## Storage of study participants’ samples

Study participants will be asked to consent to allowing any residual samples to be stored for future testing and given the option to refuse. Participants who change their mind regarding storage of their samples may contact a study investigator at any time to request that their samples be destroyed. Future use of specimens by other investigators and collaborators will be limited to their receipt as anonymized samples.

# References

1. Hallam HJ, Hallam S, Rodriguez SE, Barrett ADT, Beasley DWC, Chua A, et al. Baseline mapping of Lassa fever virology, epidemiology and vaccine research and development. NPJ Vaccines. 2018;3:11

2. WHO Target Product Profile for Lassa virus Vaccine. <https://www.who.int/blueprint/priority-diseases/key-action/LassaVirusVaccineTPP.PDF?ua=1>: World Health Organisation, June 2017.

3. Houlihan C, Behrens R. Lassa fever. 2017;358:j2986

4. Bausch DG, Demby AH, Coulibaly M, Kanu J, Goba A, Bah A, et al. Lassa fever in Guinea: I. Epidemiology of human disease and clinical observations. Vector Borne Zoonotic Dis. 2001 Winter;1(4):269-81

5. McCormick JB, Webb PA, Krebs JW, Johnson KM, Smith ES. A prospective study of the epidemiology and ecology of Lassa fever. The Journal of infectious diseases. 1987 Mar;155(3):437-44

6. World Health Organisation. An R&D Blueprint for Action to Prevent Epidemics: Plan of Action. Geneva, Switzerland: 2016.

7. Raabe V, Koehler J. Laboratory Diagnosis of Lassa Fever. Journal of clinical microbiology. 2017 Jun;55(6):1629-37

8. World Health Organisation. Lassa Fever: diagnosis 2019. Available from: <https://www.who.int/emergencies/diseases/lassa-fever/en/>.

9. Olayemi A, Obadare A, Oyeyiola A, Fasogbon S, Igbokwe J, Igbahenah F, et al. Small mammal diversity and dynamics within Nigeria, with emphasis on reservoirs of the lassa virus. Systematics and Biodiversity. 2017;16(2):118-27

10. Bausch DG. Arenaviruses. Clinical Virology, Fourth Edition2017. p. 1089-111.

11. Chatio S, Baiden F, Achana FS, Oduro A, Akazili J. Knowledge and Perceptions about Clinical Trials and the Use of Biomedical Samples: Findings from a Qualitative Study in Rural Northern Ghana. PLOS ONE. 2016;11(4):e0152854

12. Browne S, Carter T, Eckes R, Grandits G, Johnson M, Moore I, et al. A review of strategies used to retain participants in clinical research during an infectious disease outbreak: The PREVAIL I Ebola vaccine trial experience. Contemporary Clinical Trials Communications. 2018 2018/09/01/;11:50-4

13. Joseph PD, Caldwell PHY, Tong A, Hanson CS, Craig JC. Stakeholder Views of Clinical Trials in Low- and Middle-Income Countries: A Systematic Review. Pediatrics. 2016;137(2)

14. Massawe IS, Lusingu JP, Manongi RN. Community perception on biomedical research: A case study of malariometric survey in Korogwe District, Tanga Region, Tanzania. BMC Public Health. 2014 April 22;14(1):385

15. Lambe T, Rampling T, Samuel D, Bowyer G, Ewer KJ, Venkatraman N, et al. Detection of Vaccine-Induced Antibodies to Ebola Virus in Oral Fluid. Open forum infectious diseases. 2016;3(1):ofw031-ofw

16. Nokes D, Enquselassie F, Nigatu W, Vyse A, Cohen B, Brown D, et al. Has oral fluid the potential to replace serum for the evaluation of population immunity levels? A study of measles, rubella and hepatitis B in rural Ethiopia. Bulletin of the World Health Organization. 2001 (79):588–95

17. UK National Health Service (NHS). Diagnosis - HIV and AIDS 03 Apr. 2018 [Accessed : 19 Aug 2018]. Available from: <https://www.nhs.uk/conditions/hiv-and-aids/diagnosis/>.

18. Glynn JR, Bower H, Johnson S, Houlihan CF, Montesano C, Scott JT, et al. Asymptomatic infection and unrecognised Ebola virus disease in Ebola-affected households in Sierra Leone: a cross-sectional study using a new non-invasive assay for antibodies to Ebola virus. The Lancet Infectious Diseases. 2017

19. Asogun D, Tobin EA, Gunther S, Happi C, Ikponwosa O. Dealing with the unseen: Ffear and stigma in lassa fever. International Journal of Infectious Diseases. 2014;21:221

**b) PARTICIPANT CONSENT/ASSENT FORM:**

Participant ID Number:

|  | **Please circle Yes or No** | |
| --- | --- | --- |
| **CONFIRMATION** |  |  |
| - I understand that my participation in this study is voluntary and that I can stop taking part without giving any reason | Yes | No |
| - I understand that my medical care and rights will not be affected if I refuse to take part or stop taking part in this study at any time | Yes | No |
| - I understand that to participate in this study I will be asked to provide an oral fluid sample and a blood sample which will be tested in this country | Yes | No |
| - I understand that I will be asked questions about my background and health as part of this study | Yes | No |
| - I understand that my samples, including genetic material, may be stored and used in other research projects in the future, and may be shared in the future with other researchers for their ethically approved projects | Yes | No |
| - I understand that as a participant in this study, I will receive a single payment for any time, inconvenience and any transport costs incurred related to participation in this study | Yes | No |
| **CONSENT** |  |  |
| - I confirm I have read the information sheet, or that it has been explained to me. I understand the information. I have had the opportunity to ask questions about the study and have been answered satisfactorily | Yes | No |
| - I understand and give permission for authorised members of the research team to have access to information from my medical notes at Kenema Government Hospital Lassa Unit where it is relevant to this research | Yes | No |
| - I understand and give permission for my information collected during this study to be analysed, reported and shared with other researchers within and outside the country. My name will not be used and I will not be identifiable from this information. | Yes | No |
| - I agree to take part in the above named study | Yes | No |
| **FUTURE STUDIES** |  |  |
| - I give permission for my samples, including genetic material, to be used and analysed in future research projects, if necessary in another country, with ethical approval | Yes | No |
| - I give permission for researchers to contact me again to invite me to participate in future studies. | Yes | No |

|  |  |  |
| --- | --- | --- |

**Printed name of participant Signature of participant (assent if child >12) Date: dd-mmm-yyyy**

|  |  |  |
| --- | --- | --- |

**Printed name of parent/guardian for child <16y Signature of parent/guardian for child <16y Date: dd-mmm-yyyy**

|  |  |  |
| --- | --- | --- |

**Printed name of impartial witness* Signature of impartial witness* Date: dd-mmm-yyyy**

****Witnessed consent (if the consenting person cannot read the form)***

*I confirm that I have no interest or involvement in this research study, that the information about this research was accurately read and explained to the patient in language they can understand, and that informed consent was freely given by the participant*

|  |  |  |
| --- | --- | --- |

**Printed name of person obtaining consent Signature of person obtaining consent Date: dd-mmm-yyyy**

**ASSENT FORM: CHILDREN > 12 years**

**With children aged > 12 years, ask the following questions before asking them to sign the consent/assent. Assent does not replace the consent of the parent or guardian but is in addition.**

|  | **Please circle all you agree with:** | |
| --- | --- | --- |
| Have you read (or had read to you) information about this project? | Yes | No |
| Has somebody else explained this project to you? | Yes | No |
| Do you understand what this project is about? | Yes | No |
| Have you had any questions answered in a way you understand? | Yes | No |
| Do you understand that it is ok to stop taking part at any time? | Yes | No |
| Are you happy to take part? | Yes | No |

**If you would like to take part in this project, please write your name and today’s date:**

|  |  |
| --- | --- |

Your name Date

**c) Study Questionnaire**

**STUDY CHECKLIST**

| **Tick/date/initial as required when task/document is completed** | | **Notes** |
| --- | --- | --- |
| Participant ID No.: (write number) | # |  |
| Date of Visit: | dd - mmm - yyyy |  |
| Eligibility Verified | initial |  |
| Consent Form Completed | initial |  |
| Temperature recorded | initial |  |
| Questionnaire Completed | initial |  |
| Oral Fluid Collection Completed | initial |  |
| Blood Collection Completed | initial |  |
| Samples received by Study Coordinator | initial |  |
| Entered into Enrolment Log | initial |  |
| Entered into Database | dd - mmm – yyyy  initial |  |

**SURVIVOR PARTICIPANT QUESTIONNAIRE**

| **Temperature (°C)** | **If above 37.9°C, postpone sampling and recommend to visit a doctor** |
| --- | --- |

**DEMOGRAPHIC DATA**

| **Interview Date** | **Interviewer Initials** | | **Participant ID** |
| --- | --- | --- | --- |
| **Surname** | **First Name** | | **Other Names** |
| **Date of Birth:** ___ - _____ - ____ dd - mmm - yyyy  **Age** years | | | **Gender** (circle one)  **Male Female** |
| **Current Residence**  District _______________________________  Chiefdom/ Ward _______________________  Village________________________________ | | **Previous Residence**  District _______________________________  Chiefdom/ Ward _______________________  Village________________________________ | |
| **Marital Status:** Single Married Divorced Widowed | | | |
| **Cell Phone Number** | | | **Cell Phone Number of Caretaker** |
| **Occupation** (circle all that apply)  Health worker Traditional healer Traditional birth attendant Community/religious leader  Animal Farmer Crop Farmer Butcher Hunter Cook Cleaner  Labourer Trader Teacher Professional/Business Student  Child/Pupil Housewife Retired Unemployed  Other_____________________________(specify) | | | |

**OTHER MEDICAL CONDITIONS**

| Do you have any chronic illness? Yes No |
| --- |
| If Yes, name of illness(es), or main symptoms |
| Are you an Ebola survivor? Yes No |
| If Yes, please provide date of discharge |
| During the last 12 months have you been diagnosed with Malaria? Yes No |
| Are you currently pregnant? Yes No  Have you given birth to a child within the last year? Yes No |

**LASSA FEVER EXPOSURE SINCE DISCHARGE**

| **Do you think you may have been sick with Lassa Fever again since you were discharged from hospital?**  **Yes No** | |
| --- | --- |
| **If Yes, please describe symptoms and dates** | |
| **If yes, how do you think you were infected in this new illness?**  (circle one) | 1. Contact with rodents or rodent droppings in or near your household/compound 2. Contact with rodents or rodent droppings in another location 3. Eating contaminated food 4. Hunting rodents/small animals 5. Preparing rodents/small animals for cooking 6. Caring for someone who was ill 7. Contact with someone who was ill but not in your care 8. Witchcraft / Curse 9. Other (describe) 10. Don’t know |
| Has anyone within your household had Lassa Fever since you were discharged from hospital?  Yes No | |
| If Yes, please describe symptoms and dates | |
| Do you know anyone outside your household who got Lassa Fever since you were discharged?  Yes No | |
| If Yes, did you have contact with them?  Yes No | |
| If Yes, please describe symptoms and dates of the person who was ill | |
| Have you made any changes in your household or practices to prevent Lassa infections since you were discharged from hospital?  Yes No | |
| If Yes, please describe | |

**LASSA FEVER HISTORY**

| **Duration of symptoms before admission to hospital?**  1-3 days 4-7 days 8-14 days 15-21 days 22-28 days >28 days | |
| --- | --- |
| **Symptoms at admission?**  (Circle all numbers that apply) | |
| 1. Fever | 1. Muscle and/or joint pain/ache |
| 1. Fatigue (general body weakness) | 1. Sore throat or pain with swallowing |
| 1. Headache | 1. Hiccups |
| 1. Loss of appetite | 1. Red eyes |
| 1. Nausea or Vomiting | 1. Blurry vision |
| 1. Abdominal pain | 1. Bleeding gums |
| 1. Diarrhoea | 1. Miscarriage |
| 1. Blood in the stool | 1. Other (please describe) |
| **Severity of illness at admission** (Circle one): Mild Moderate Severe | |
| **Do you think you know how you got sick?**  (circle one) | 1. Contact with rodents or rodent droppings in or near your household/compound 2. Contact with rodents or rodent droppings in another location 3. Eating contaminated food 4. Hunting rodents/small animals 5. Preparing rodents/small animals for cooking 6. Caring for someone who was ill 7. Contact with someone who was ill but not in your care 8. Witchcraft / Curse 9. Other (describe) 10. Don’t know |
| **When were you discharged from hospital?** ___ - _____ - ____ **dd – mmm - yyyy** | |
| **Laboratory results on discharge** | |

**PERCEPTION OF ORAL SAMPLE COLLECTION**

| Do you prefer giving a sample using the oral swab or do you prefer blood sample collection?  Oral Fluid Blood sample |
| --- |
| Do you think you would be happy to give an oral fluid sample every 2-4 weeks if you were part of a research study? Yes No |
| Any other comments/feedback about using the oral swab? |

**CONTROL PARTICIPANT ELIGIBILITY CHECK**

**For ‘Not eligible’ responses: do not recruit into the study; no further questions, no sample collection.**

| **Were you ever tested for Lassa Fever?** (circle one) **Yes No** | |
| --- | --- |
| **What was the final result of your test for Lassa Fever?** (circle one)  **Negative**  **Didn’t receive result (Not eligible) Don’t remember (Not eligible) Positive (Not eligible)** | |
| **Have you ever lived, worked or visited in a Lassa-endemic area? Yes (Not eligible) No** | |
| **Has anyone within your household had Lassa Fever? Yes (Not eligible) No** | |
| **Do you know anyone outside your household who got Lassa Fever? Yes No** | |
| **If Yes, did you have contact with them? Yes (Not eligible) No** | |
| **Temperature: (°C)** | **If above 37.5°C – Not eligible for study participation, no further questions and no sample collection. Advise individual to seek medical advice** |

**CONTROL PARTICIPANT QUESTIONNAIRE**

**DEMOGRAPHIC DATA**

| **Interview Date** | **Interviewer Initials** | | **Participant ID** |
| --- | --- | --- | --- |
| **Surname** | **First Name** | | **Other Names** |
| **Age _______** years **Date of Birth:** ___ - _____ - ____  dd - mmm - yyyy | | | **Gender** (circle one)  **Male Female** |
| **Current Residence:**  District _______________________________  Chiefdom/ Ward _______________________  Village________________________________ | | **Previous Residence:**  District _______________________________  Chiefdom/ Ward _______________________  Village________________________________ | |
| **Marital Status:** Single Married Divorced Widowed | | | |
| **Cell Phone Number:** | | | **Cell Phone Number of Caretaker:** |
| **Occupation** (circle all that apply)  Health worker Traditional healer Traditional birth attendant Community/religious leader  Animal Farmer Crop Farmer Butcher Hunter Cook Cleaner  Labourer Trader Teacher Professional/Business Student  Child/Pupil Housewife Retired Unemployed  Other_____________________________(specify) | | | |

**OTHER MEDICAL CONDITIONS**

| Do you have any chronic illness? Yes No |
| --- |
| If Yes, name of illness(es), or main symptoms |
| Are you an Ebola survivor? Yes No |
| If Yes, please provide date of discharge |
| During the last 12 months have you been diagnosed with Malaria? Yes No |
| Are you currently pregnant? Yes No  Have you given birth to a child within the last year? Yes No |

**PERCEPTION OF ORAL SAMPLE COLLECTION**

| Do you prefer giving a sample using the oral swab or do you prefer blood sample collection?  Oral Fluid Blood sample |
| --- |
| Do you think you would be happy to give an oral fluid sample every 2-4 weeks if you were part of a research study? Yes No |
| Any other comments/feedback about using the oral swab? |

**d) Survivor and Control Patient Information Sheets**

**Lassa Virus Survivor Participant Information Sheet**

**My name is xxx and I am working with the UK Public Health Rapid Support Team and Kenema Government Hospital. We are carrying out a study to understand whether we can find immune responses to Lassa fever in oral fluids.** The study has been approved by the Sierra Leone Ethics and Scientific Review Committee and the London School of Hygiene & Tropical Medicine Ethics Committee.

**You/your child are being invited to take part in this research study. Before you decide to take part, it is important for you to understand why the research is being done and what it will involve. Please read this information about this study, or I can read it to you. Please ask me if there is anything that is not clear or if you would like more information.**

**What are we trying to learn with this research study?**

Lassa Fever can cause severe illness in adults and children. We know that many people who have had Lassa fever develop ‘antibodies’ - a protection in their body - that lasts a long time after they recover. Usually we measure this protection by taking blood, but we think it could be measured in the “oral fluid” found in the mouth and that this would be easier for patients, health-workers and researchers. We have developed a new method to test oral fluid and we are asking you to give an oral fluid and a blood sample so that we can test whether this new method can measure the Lassa Fever antibodies accurately and whether it is as good as, or better than, using blood. If we find this is true, we

and other researchers would be able to ask for oral fluids samples instead of blood samples to better understand Lassa fever and Lassa virus transmission.

**Why are we asking you to participate?**

We are asking people who have had Lassa Fever and who were treated at the Kenema Government Hospital to take part in the study. We think you may have the antibodies we are looking for in your blood and oral fluids.

**What happens if I don’t want to participate in the study?**

You are free to refuse to participate in this study, or to withdraw at any time. Refusal to participate or withdrawal will not affect you/your child in any way.

**What will my participation in this study involve?**

If you choose to participate in this study, we will ask you for about one hour of your time. We will also ask you if you are happy for us to contact you again in future. If you agree to take part, we will first take your body temperature. If your temperature is 37.5^o^C or above, we will suggest that you visit the doctor as this may be a sign of an illness and we will not continue with the study.

If your temperature is 37.5^o^C or below, we will ask you to sign a form giving your consent to take part, or your consent for your child to take part. Then we will ask you some questions about who you are, about your general health, and about your exposure to things that cause Lassa fever. We will ask you/your child to give an oral fluid sample and we will take a 5ml blood sample. All samples will be analysed in Sierra Leone. We will also ask you for permission to look at the medical records from when you/your child were treated for Lassa fever at Kenema General Hospital so that we can see how the antibodies we measure are related to the illness.

**If you/your child are found to have antibodies to lassa virus what does it mean?**

If your sample is positive, this relates to the Lassa fever or Lassa virus infection(s) you had in the past. It does not mean that you have Lassa fever or a Lassa virus infection now. It does not mean that you are infectious. However, because we cannot say that a positive test means you are immune (protected) against Lassa virus and you might still be able to get Lassa virus again, we will not tell you the result of the test as there is no benefit in knowing this.

**What happens to the blood and oral fluid samples?**

We would like to store your/your child’s samples for possible later testing, for example if more accurate tests for Lassa Fever or other ways to investigate antibodies become available. If you do not want your samples to be stored, you can tell us and we will not store them, and if you change your mind in the future, you can tell us to destroy the stored samples. If we want to do further tests on the stored samples we will ask for permission first from the Sierra Leone Ethics & Scientific Review committee.

**Are there any risks involved with participating in this study?**

There are no direct risks from participating in this study. There is a small risk of pain or irritation when samples are taken. Some of the questions we ask may remind you of painful events, such as when you were sick. If these questions are too difficult for you to answer, you are free to stop the interview at any time or leave the study completely.

**Are there any benefits involved with participating in this study?**

There will be no direct benefits from participating in this study, but you will be provided with a small amount of money for any inconvenience and transport costs.

**Will I be allowed to withdraw from the study if I change my mind?**

Taking part in this study is voluntary. You should join this study only if you want to and only if you are happy to give an oral fluid and blood sample. Even if you agree to give the samples, you can choose not to answer some or all of the questions. You can leave the study at any time and it will not affect you in any way. Not participating will not affect any health care that you might need in the future or your participation in any other research study.

**Who will see the information that is collected?**

Your name and other identifying information will be removed from the questionnaire before analysis, and all data will be stored in a way that only authorised people can access it. Your personal information will not be revealed in any published information.

We are happy to discuss any worries or queries. If you have any questions during the study or want any information, please contact:

The Study Coordinator on xx xxx xxxx

The Principal Investigator on xx xxx xxxx

Thank you for your time and for considering this study.

**Lassa Virus Control Participant Information Sheet**

**My name is xxx and I am working with the UK Public Health Rapid Support Team and Kenema Government Hospital. We are carrying out a study to understand whether we can find immune responses to Lassa fever in oral fluids.** The study has been approved by the Sierra Leone Ethics and Scientific Review Committee and the London School of Hygiene & Tropical Medicine Ethics Committee.

**You are being invited to take part in this research study. Before you decide to take part, it is important for you to understand why the research is being done and what it will involve. Please read this information about this study. Please ask me if there is anything that is not clear or if you would like more information.**

**What are we trying to learn with this research study?**

Lassa Fever can cause severe illness in adults and children. We know that many people who have had Lassa fever develop ‘antibodies’ - a protection in their body - that lasts a long time after they recover. Usually we measure this protection by taking blood, but we think it could be measured in the “oral fluid” found in the mouth and that this would be easier for patients, health-workers and researchers. We have developed a new method to test oral fluid and we are asking you to give an oral fluid and a blood sample so that we can test whether this new method can measure the Lassa Fever antibodies accurately and whether it is as good as, or better than, using blood. If we find this is true, we and other researchers would be able to ask for oral fluids samples instead of blood samples to better understand Lassa fever and Lassa virus transmission.

**Why are we asking you to participate?**

We are asking you to participate because we want to look at immune responses in people who have not had Lassa fever or have not been exposed to Lassa virus, in order to compare these samples with samples from Lassa fever survivors. We think you will not have antibody responses to Lassa virus in your blood because you live and work in an area outside the Lassa virus endemic area in Sierra Leone. We have permission from the college to ask you to participate but it is your choice to take part.

**What happens if I don’t want to participate in the study?**

You are free to refuse to participate in this study, or to withdraw at any time. Refusal to participate or withdrawal will not affect you in any way.

**What will my participation in this study involve?**

If you choose to participate in this study, we will first ask you some questions to understand whether you may have been exposed to Lassa virus, and we will take your body temperature. If we think you may have been exposed to Lassa virus, we will not recruit you because for this part of the study we are looking for people who have not been exposed. If your body temperature is 37.5^o^C or above, we will not recruit you to the study and will suggest that you to seek medical advice.

If we are confident that you haven’t been exposed to Lassa virus, and your body temperature is 37.5^o^C or below, we will ask you to participate in the study. We will ask you to sign a form giving your consent to take part. Then we will ask you some questions about who you are and about your general health. We will ask you to give an oral fluid sample and a 5ml blood sample. All samples will be analysed in Sierra Leone.

**If you are found to have antibodies to lassa virus what does it mean?**

If your sample is positive, this relates to the Lassa fever or Lassa virus infection(s) you have had in the past. It does not mean that you have Lassa fever or a Lassa virus infection now. It does not mean that you are infectious. However, because we cannot say that a positive test means you are immune (protected) against Lassa virus and you might still be able to get Lassa virus again, we will not tell you the result of the test as there is no benefit in knowing this.

**What happens to the blood and oral fluid samples?**

We would like to store your samples for possible later testing, for example if more accurate tests for Lassa Fever or other ways to investigate antibodies become available. If you do not want your samples to be stored, you can tell us and we will not store them. If you change your mind in the future, you can tell us to destroy the stored samples. If we want to do further tests on the stored samples we will ask for permission first, from the Sierra Leone Ethics and Scientific Review Committee.

**Are there any risks involved with participating in this study?**

There are no direct risks from participating in this study. There is a small risk of pain or irritation when samples are taken. Some of the questions we ask may remind you of painful events, such as when you or others were sick. If these questions are too difficult for you to answer, you are free to stop the interview at any time or leave the study completely.

**Are there any benefits involved with participating in this study?**

There will be no direct benefits from participating in this study, but you will be provided with a small amount of money for any inconvenience and transport costs. We have also arranged with the Dean of your college to provide lectures on Lassa Fever and running a research study which will be open to all students.

**Will I be allowed to withdraw from the study if I change my mind?**

Taking part in this study is voluntary. You should join this study only if you want to and only if you are happy to give an oral fluid and blood sample. Even if you agree to give the samples, you can choose not to answer some or all of the questions. You can leave the study at any time and it will not affect you in any way. Not participating will not affect any health care that you might need in the future or your participation in any other research study. Participation is not a requirement of your study and not taking part will not affect any aspect of your study.

**Who will see the information that is collected?**

Your name and other identifying information will be removed from the questionnaire before analysis, and all data will be stored in a way that only authorised people can access it. Your personal information will not be revealed in any published information.

We are happy to discuss any worries or queries. If you have any questions during the study or want any information, please contact:

The Study Coordinator on xx xxx xxxx,

The Principal Investigator on xx xxx xxxx

Thank you for your time and for considering this study.
