# Supplementary material for: Boosting understanding of Lassa Fever virus epidemiology: Field testing a novel assay to identify past Lassa Fever virus infection in blood and oral fluids of survivors and unexposed controls in Sierra Leone
Source: PLoS Negl Trop Dis. 2021 Mar 31;15(3):e0009255. doi: 10.1371/journal.pntd.0009255 (PMC8041174; doi:10.1371/journal.pntd.0009255)
Supplement: S1 Table — (DOCX) [file pntd.0009255.s004.docx]

| **S1 Table. Symptoms at admission recalled by survivors at time of recruitment (n=71)** | | |
| --- | --- | --- |
| Fever | 65 | 91.5% |
| Headache | 45 | 63.4% |
| Loss of appetite | 35 | 49.3% |
| Nausea or vomiting | 27 | 38.0% |
| Fatigue | 28 | 39.4% |
| Red eyes | 25 | 35.2% |
| Diarrhoea | 16 | 22.5% |
| Sore throat/ pain with swallowing | 11 | 15.5% |
| Miscarriage (% of women) | 4 | 5.6% |
| Hiccups | 7 | 9.9% |
| Muscle and/or joint pain/ache | 6 | 8.5% |
| Abdominal pain | 7 | 9.9% |
| Blurry vision | 4 | 5.6% |
| Blood in stool | 3 | 4.2% |
| Dizziness | 2 | 2.8% |
| Cough | 2 | 2.8% |
| Bleeding gums | 2 | 2.8% |
| Blood in urine | 1 | 1.4% |
| Nosebleed | 1 | 1.4% |
| Vaginal bleeding | 1 | 1.4% |
| Rash | 1 | 1.4% |
| Other (swollen lips, warm neck, other) | 3 | 4.2% |
